# Dipnictogen Radical Chemistry: A Dithorium-Supported Distibene Radical Trianion

**DOI:** 10.1021/jacs.4c15431

**Published:** 2025-01-23

**Authors:** Jingzhen Du, Kevin Dollberg, John A. Seed, Floriana Tuna, Ashley J. Wooles, Carsten von Hänisch, Stephen T. Liddle

**Affiliations:** † Department of Chemistry and Centre for Radiochemistry Research, The University of Manchester, Oxford Road, Manchester M13 9PL, U.K.; ‡ Fachbereich Chemie, Philipps-Universität Marburg, Hans-Meerwein-Straße 4, Marburg 35043, Germany; § Department of Chemistry and Photon Science Institute, The University of Manchester, Oxford Road, Manchester M13 9PL, U.K.

## Abstract

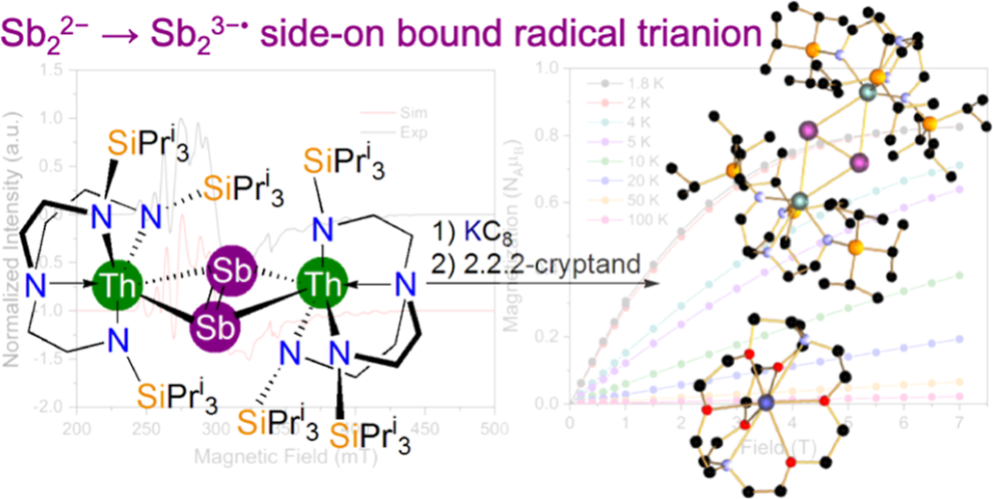

Although two examples of σ-bonded *trans*-bent [RSbSbR]^•–^ (R = bulky organo- or Ga-groups) that formally contain the Sb_2_^•3–^ radical trianion moiety are known in p-block chemistry, d- or f-element Sb_2_^•3–^ radical trianion complexes, with or without R-substituents, have remained elusive. Here, we report that reduction of a 77:23 mix of [{Th(Tren^TIPS^)}_2_(μ-η^2^:η^2^-Sb_2_)] (**3a**, Tren^TIPS^ = {N(CH_2_CH_2_NSiPr^i^_3_)_3_}^3–^):[{Th(Tren^TIPS^)}_2_(μ-SbH)] (**3b**) with 1.5 equiv of KC_8_ in the presence of 1.1 equiv of 2.2.2-cryptand yields the emerald green Sb_2_^•3–^ radical complex [K(2.2.2-cryptand)][{Th(Tren^TIPS^)}_2_(μ-η^2^:η^2^-Sb_2_)] (**4**), providing an f-block Sb_2_^•3–^ radical trianion complex, and the heaviest actinide-N_2_ radical analogue. When the recrystallization conditions are modified, a small crop of red crystals determined to be [K(2.2.2-cryptand)]_3_[{Th(Tren^TIPS^)(μ-η^3^:η^3^-Sb_3_)}_2_(μ-K)] (**5**) were also isolated, highlighting the complexity of heavy group 15 homodiatomic reduction chemistry. SQUID magnetometry and EPR spectroscopy suggest that the Sb_2_^•3–^ radical trianion in **4** is fairly well isolated, due to electrostatic binding to Th, with pseudoaxial *g*-values reflecting the distinctive Sb_2_^•3–^ radical trianion side-on bridging π-bonded coordination mode. Spectroscopically validated computational analysis of **3a** and **4** confirms the stronger donating capability, and weaker Sb–Sb bond, of Sb_2_^•3–^ radical trianion compared to the Sb_2_^2–^ dianion form.

## Introduction

Homo- and heterodiatomic radicals such as O_2_^•–^ and NO^•^ have played a foundational role in developing basic chemical concepts and are relevant to biological and industrial processes.^1−6^ Likewise, reduced group 15 homodiatomics, epitomized by N_2_^*n*^ (*n* = 0, −1, −2, −3, −4) are fundamentally important not only because they are key intermediates in the conversion of N_2_ to ammonia^7−12^ but also since it was discovered that the N_2_^•3–^ radical trianion can support remarkable magnetic properties in rare earth complexes^13−15^ and play a role in actinide (An)-mediated N_2_ cleavage to nitrides.^16−18^

There has been growing interest in studying new, heavier homologues of Pn_2_ (Pn = P, As, Sb, Bi) and in particular open shell radicals due to their novel electronic structures.^19^ Significant advances have been disclosed, evidenced by the isolation of molecular P_2_^*n*^ (*n* = +2, +1, 0, −1, −2, −3, −4),^20−36^ As_2_^*n*^ (*n* = +1, 0, −2, −4),^37−48^ Sb_2_^*n*^ (*n* = 0, −2, −3),^49−57^ and Bi_2_^*n*^ (*n* = −2, −3) complexes,^58−61^ but they are mainly stabilized by d- and p-block metal ions or sterically demanding organo-substituents. In contrast, f-element Pn_2_ derivatives, and especially their radical forms, are exceedingly rare.^19^ Although lanthanide-Sb_2_^2–^ and -Bi_2_^*n*^ (*n* = −2, −3) complexes are known,^53,59,61^ they have been elusive for the An-ions, and Sb_2_ radicals of any type remain unknown as d- or f-block derivatives. There are hints that binary Cs_3_Sb_2_ has been prepared,^62^ but characterization is limited to conductivity measurements,^62^ so whether Cs_3_Sb_2_ is that, or [Cs_3_Sb_2_][e], as is the case with [M_3_Bi_2_][e] (M = K, Rb, Cs), is unknown.^63^ Compared with N, P, and As, Sb is softer with more metal character, so it is a poor match for hard An-ions. Hence, installing radical character would impose additional difficulties in terms of synthesis and isolation. Indeed, actinide-Sb and -Bi bonds remain rare generally.^19,57,64−68^

A limiting factor in this area is the lack of suitable precursors and routes for constructing Sb_2_ units. However, we recently reported that reaction of [K(18C6)(THF)SbH_2_] (**1**, 18C6 = 18-crown-6 ether)^69^ with [Th(Tren^TIPS^)(DME)][BPh_4_] (**2**, Tren^TIPS^ = {N(CH_2_CH_2_NSiPr^i^_3_)_3_}^3–^, DME = 1,2-dimethoxyethane)^70^ generated a 77:23 mix of [{Th(Tren^TIPS^)}_2_(μ-η^2^:η^2^-Sb_2_)] (**3a**):[{Th(Tren^TIPS^)}_2_(μ-SbH)] (**3b**).^57^ Reduction of a mixture of **3a**:**3b** produced the stibido tetramer [{Th(Tren^TIPS^)(μ-SbK_2_)}_4_].^57^ Since the K^+^ cations evidently stabilize the tetramer, we surmised that sequestering them during the reduction could disfavor Sb_2_ cleavage and possibly enable a Sb_2_^•3–^ radical trianion intermediate to be isolated. Such a species would be unique because there are only two examples of crystallographically authenticated Sb_2_-derived radicals, namely, [Li(DME)_3_][RSbSbR] (R = C_6_H_2_-2,6-(CH(SiMe_3_)_2_)_2_-4-C(SiMe_3_)_3_)^49^ and [K(B18C6)(DME)][{HC(CMeNAr)_2_}Ga(NMe_2_)Sb]_2_ (Ar = 2,6-diisopropylphenyl; B18C6 = benzo-18-crown-6 ether);^71^ both exhibit σ-bonded *trans*-bent [RSbSbR]^•–^ geometries (R = bulky organo- or Ga-groups) that can be categorized as zigzag “end-on” stabilized.

Here, we report an f-block-stabilized Sb_2_^•3–^ radical trianion complex—the heaviest actinide-N_2_ radical analogue—where the side-on coordination mode of the radical is distinct to prior zigzag “end-on” forms. Given the polarized nature of An-ligand bonding, this presents the opportunity to probe the electronic structure of the Sb_2_^•3–^ radical trianion with modest coordination/substitution effects where the pseudoaxial character of the Sb_2_^•3–^ radical trianion is evidenced.

## Results and Discussion

### Synthesis

**Scheme 1:**
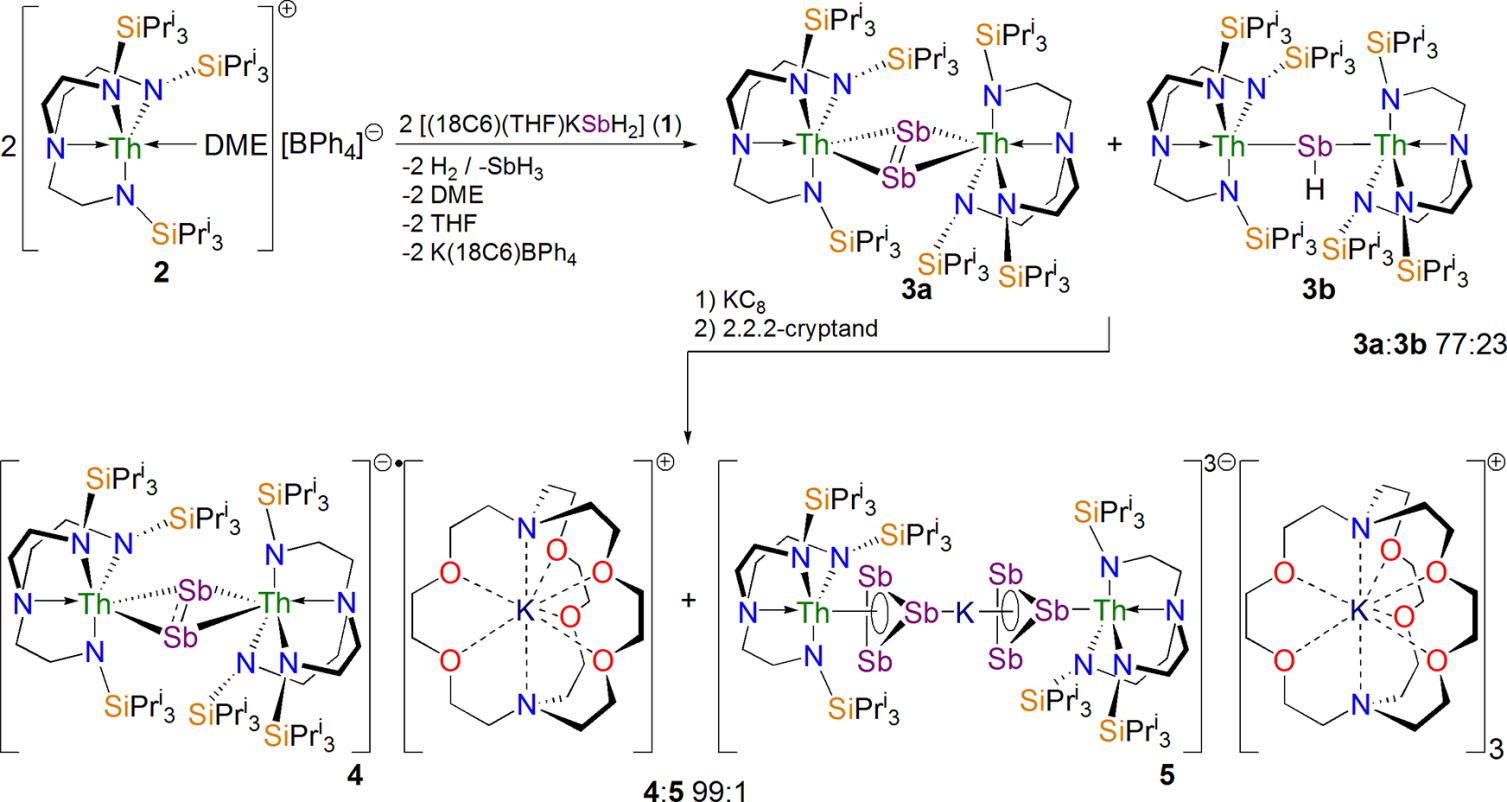
Synthesis of **4** and **5** The salt elimination reaction between **1** and **2** promotes dehydrocoupling to produce a 77:23 mixture of **3a**/**3b** which co-crystallize.^57^ Reduction of **3a**/**3b** using KC_8_ and 2.2.2-cryptand produces **4** and **5**. When the recrystallization is from THF/Et_2_O, only **4** is isolated, but if the recrystallization is done with THF/pentane, then a very small quantity of **5** is isolated along with **4**.^72^

Treatment of a 77:23% mixture of **3a**:**3b** in benzene with 1.5 equiv of KC_8_ in the presence of 1.1 equiv of 2.2.2-cryptand formed a dark green suspension. After workup and recrystallization from THF/Et_2_O, emerald green crystals of [K(2.2.2-cryptand)][{Th(Tren^TIPS^)}_2_(μ-η^2^:η^2^-Sb_2_)] (**4**) were obtained in 61% isolated yield,^72^Scheme 1. Complex **4** can be repeatedly and reliably obtained this way, but THF/pentane recrystallization affords a small quantity (<1%) of [K(2.2.2-cryptand)]_3_[{Th(Tren^TIPS^)(μ-η^3^:η^3^-Sb_3_)}_2_(μ-K)] (**5**) as red crystals with **4** still the majority crystalline product,^72^Scheme 1. Unfortunately, despite exhaustive attempts, **5** could not be synthesized on scale nor in pure form and so was not characterized beyond its solid-state crystal structure (see later) due to insufficient material, but it is reminiscent of [{U(Tren^TIPS^)}_2_{Sb_3_(μ_3_-Li)(μ-Li[THF]_2_)_3_Sb_3_}],^67^ which also contains an An(Sb_3_)M(Sb_3_)An core. This suggests that while the reduction of **3a**/**3b** is not exclusive for the formation of **4**, the Tren^TIPS^ ligands provide sufficient protection to render **4** the major product. Then, the Sb_2_ unit being retained upon reduction rather than cleaved, cf. [{Th(Tren^TIPS^)(μ-SbK_2_)}_4_],^57^ is notable, since diatomic radicals, especially ones with large negative charges, tend to be reactive, and we suggest this reflects the lack of stabilizing K^+^ cations since these are efficiently sequestrated by the 2.2.2-cryptand.^57^

### Solid-State Structures

**Figure 1:**
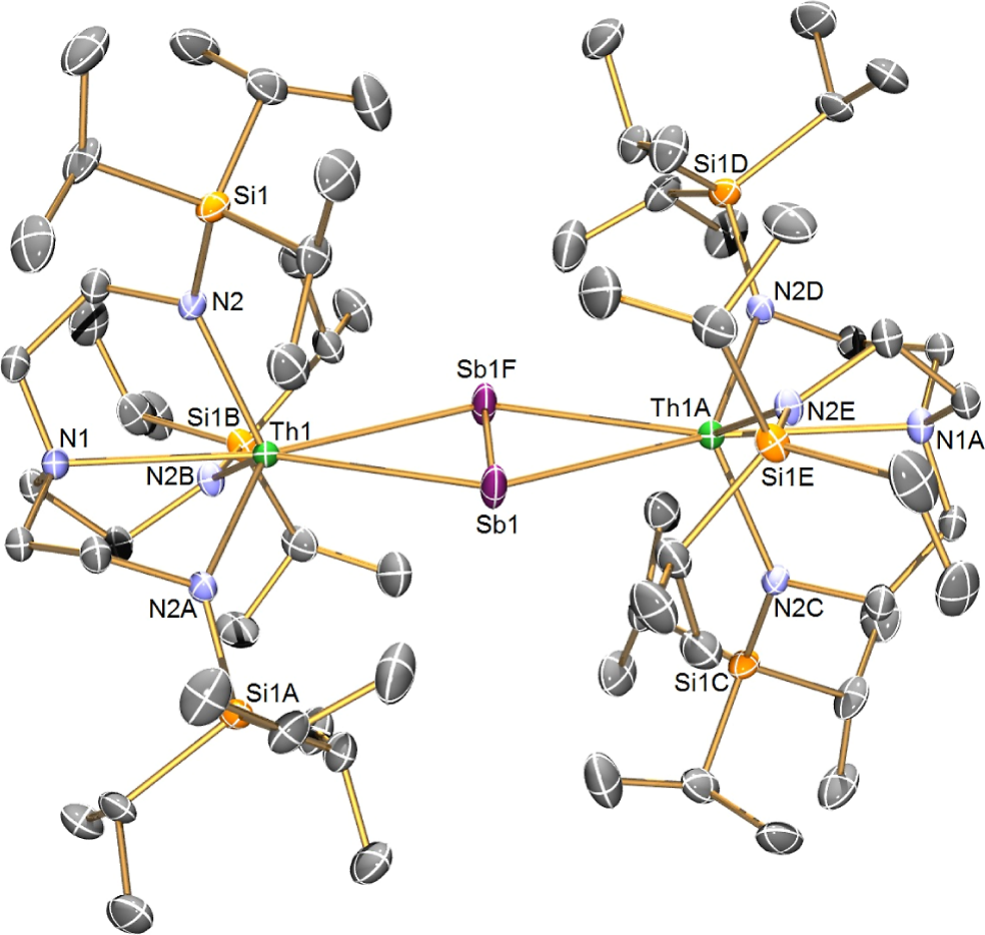
Molecular structure of the anion component of **4** (**4′**) at 100 K and with displacement ellipsoids set to 40% probability. Hydrogen atoms, disorder components, and the [K(2.2.2-cryptand)]^+^ cation component are omitted for clarity.

The solid-state structure of **4** confirms the separated ion pair formulation and, on charge-balance grounds, the presence of a Sb_2_^•3–^ radical trianion, as shown in Figure 1. In **4** the Th–Sb distances are 3.288(9)/3.313(9) Å, ∼0.15 Å longer than the sum of the covalent single bond radii of Th and Sb (3.15 Å)^73^ reflecting the side-on π-bonded coordination mode of the distibene, but is ∼0.12 Å shorter than in **3a** consistent with increased charge on Sb_2_^•3–^ compared to Sb_2_^2–^, rendering the former a stronger donor. In contrast, the Sb–Sb distance in **4** [2.796(5) Å] is longer than **3a** [2.6397(14)/2.668(8) Å] and in the complex [{Sm(Cp*)_2_}_2_(Sb_2_)] [2.6593(15) Å].^53^ That is an increase of ∼0.14 Å reflecting the presence of extra π* electron density in Sb_2_^•3–^ compared to Sb_2_^2–^ leading to a reduced Sb–Sb bond order. Indeed, the sum of the single, double, and triple bond radii of two Sb atoms are 2.80, 2.66, and 2.54 Å, respectively,^73^ so the Sb–Sb distance in **4** is, as expected, intermediate to the single and double bond metrical benchmarks due to the additional antibonding electron in **4** compared to **3a** (whose Sb–Sb distance is in-line with the presence of a formal Sb=Sb double bond). The Sb–Sb distance in **4** compares well to the Sb–Sb distances of 2.7511(4) Å in aforementioned [Li(DME)_3_][RSbSbR]^49^ and 2.7359(3) Å in [K(B18C6)(DME)][{HC(CMeNAr)_2_}Ga(NMe_2_)Sb]_2_.^71^ The Th–N_amide_ distances in **4** are statistically indistinguishable to those in **3a**,^57^ but in **4**, the Th–N_amine_ distance [2.806(12) Å] is significantly longer compared to **3a** [2.729(5) Å],^57^ reflecting the charge-rich nature of the [{Th(Tren^TIPS^)}_2_(μ-η^2^:η^2^-Sb_2_)]^•–^ unit in **4**.

**Figure 2:**
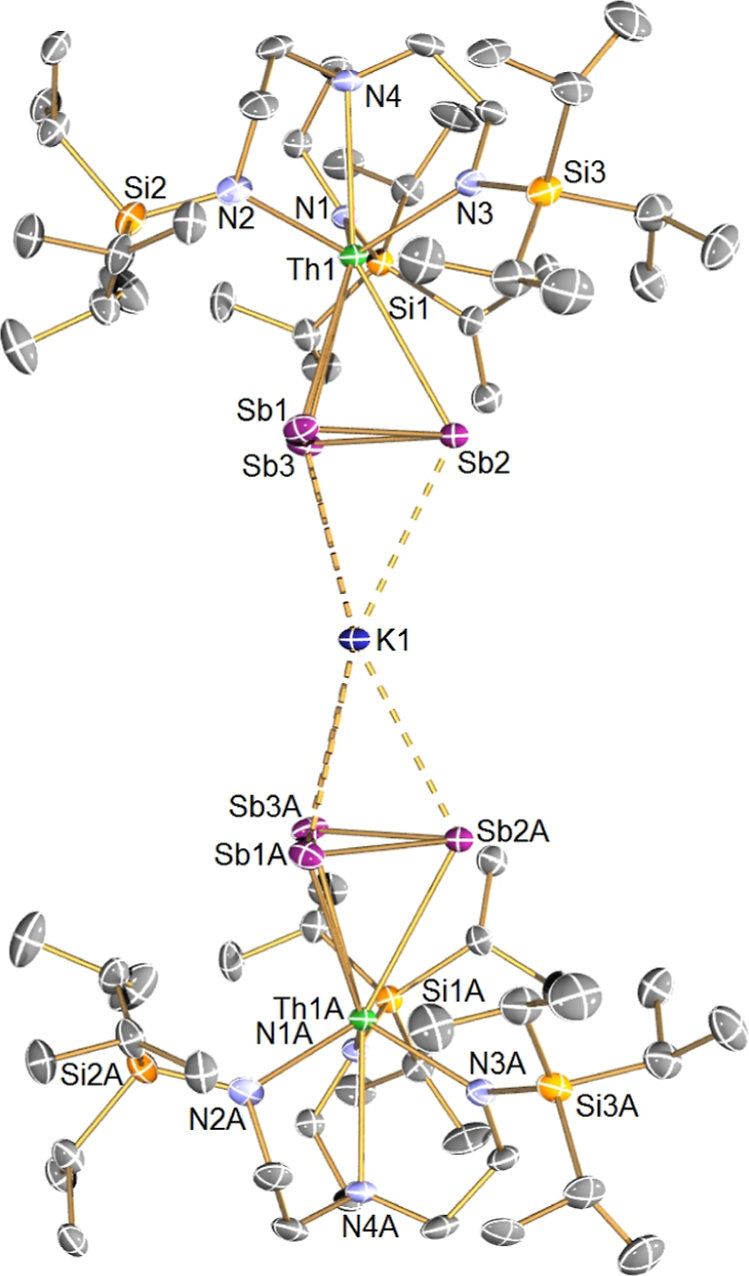
Molecular structure of the trianion component of **5** (**5′**) at 150 K and with displacement ellipsoids set to 40% probability. Hydrogen atoms and the three [K(2.2.2-cryptand)]^+^ cation components are omitted for clarity.

Although **5** is isolated in only very low yield, its solid-state structure merits further comment, Figure 2, where the trianion component of **5**, **5′** can be regarded as a sandwich complex of a K^+^ cation ligated by two {(Tren^TIPS^)Th(μ-η^3^:η^3^-Sb_3_)}^2–^ metallo-ligands. As stated earlier, the core Th(Sb_3_)K(Sb_3_)Th motif is analogous to the core U(Sb_3_)Li(Sb_3_)U of [{U(Tren^TIPS^)}_2_{Sb_3_(μ_3_-Li)(μ-Li[THF]_2_)_3_Sb_3_}],^67^ where the three K^+^ ions that are equivalent to the {Li[THF]_2_}^+^ units are sequestered by 2.2.2-cryptands. In the U version of **5**, the (Sb_3_)–(Sb_3_) units were found to weakly interact with each other,^67^ but such interactions can be ruled out in **5** due to (Sb_3_)···(Sb_3_) separations of ≥6.32 Å. The Th–Sb distances span the narrow range 3.2259(7)–3.2287(7) Å, which is similar to the Th–Sb distances in **4** and longer than Th–Sb σ-/π-bonds^57^ reflecting the π-donor character of the Sb_3_^3–^ ligands in **4**. The Th–N_amide_ [2.377(8)–2.391(10) Å] and Th–N_amine_ [2.847(8) Å] distances are long for Th–Tren complexes,^57^ reflecting the large, negative charge of the **5′** component. The K–Sb distances [3.5962(18)–3.6995(15) Å] are longer than the sum of the single covalent bond radii of K and Sb (3.36 Å),^73^ again reflecting the π-bonding and electron rich nature of **5′**.

### Spectroscopic and Magnetic Analysis

The ^13^C{^1^H} and ^29^Si{^1^H} NMR spectra (Figures S3–S6) of **4** are unexceptional, suggesting that the Sb_2_^•3–^ radical trianion does not significantly affect their chemical shifts. Likewise, the ^1^H NMR (Figures S1 and S2) chemical shifts for the [K(2.2.2-cryptand)]^+^ component in **4** are as expected. However, the ^1^H NMR chemical shifts of the Tren^TIPS^ ligand in **4** are broad and paramagnetically shifted, spanning 7 to −6 ppm, consistent with the radical nature of **4**.

The IR and Raman spectra of **4** (Figures S7 and S8) were collected and found to be consistent with the computed analytical frequencies calculation on the anion of **4**, **4′** (Figure S9). For example, **4** exhibits inelastic scattering bands at 71, 104, and 221 cm^–1^, assigned as Th–Sb, Th–Sb, and Sb–Sb stretches from the analytical frequencies calculation which predicts them to occur at 67, 111, and 209 cm^–1^, respectively. The corresponding bands for **3a** are 66, 105, and 239 cm^–1^, and although the differences are modest, the data are consistent with stronger Th–Sb and weaker Sb–Sb bonds in **4** compared to those in **3a**, in-line with the crystallographic metrical data.

**Figure 3:**
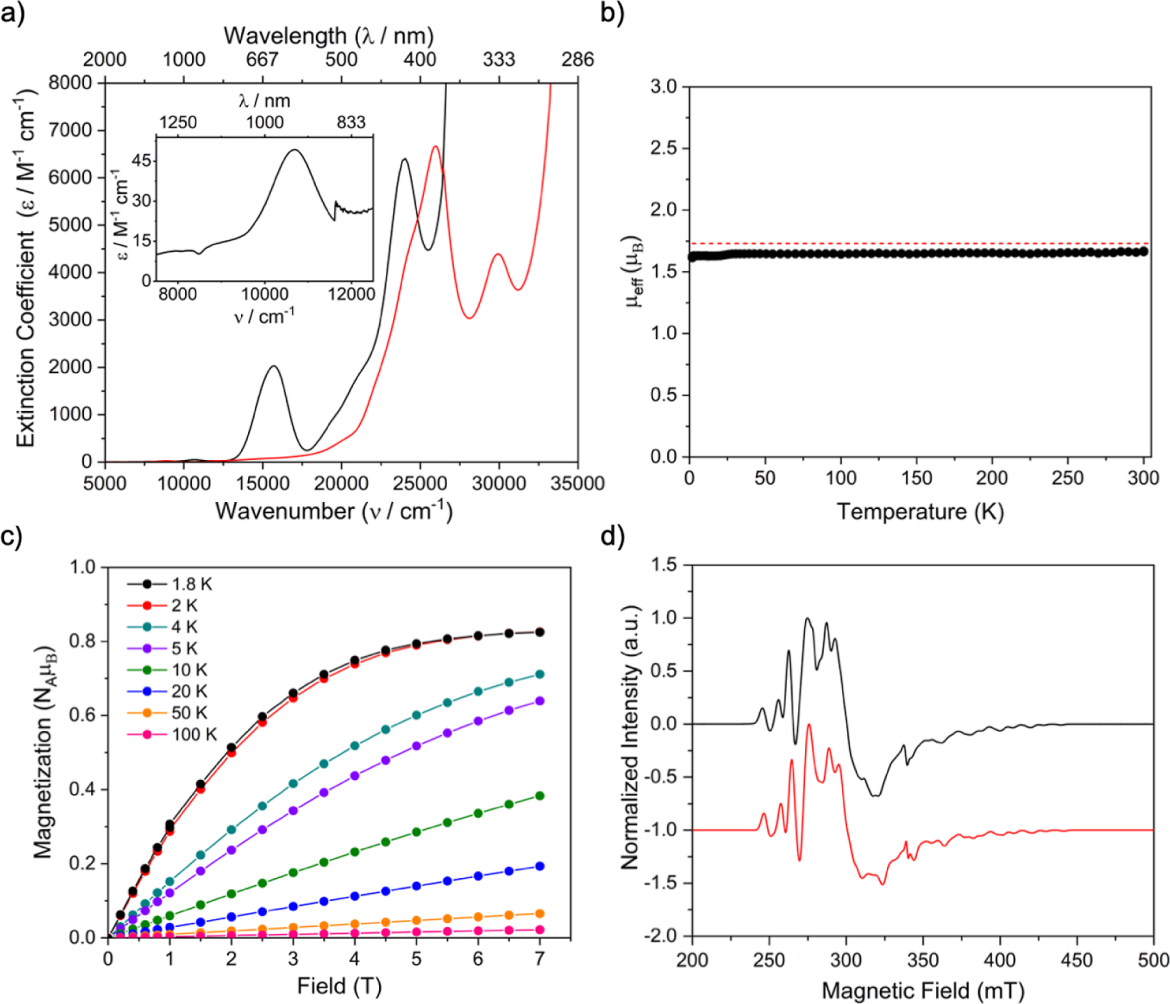
Characterization data for **4**. (a) UV/vis/NIR spectrum of **4** (black line, inset of 7500–12,500 cm^–1^ region to highlight the weak HOMO–LUMO absorption at 10,718 cm^–1^) with **3a** for comparison (red line). (b) Variable-temperature SQUID magnetometry effective magnetic moment (μ_eff_, μ_B_) of powdered **4** (black dots) with the hypothetical value of a *S* = 1/2 (*g* = 2) single unpaired electron (red dashes). (c) Magnetization (N_A_μ_B_) vs field (T) of **4** at temperatures specified by the key. (d) Experimental (black line) and simulated (red line) X-band EPR spectra of polycrystalline **4**, with *g*_*x*_ = 2.29, *g*_*y*_ = 2.26, *g*_*z*_ = 1.97 (*g*_iso_ = 2.18) and *A*(^121/123^Sb) of 116, 162, and 538 MHz (^121/123^Sb isotopes: *I* = 5/2, 57.25%; 7/2, 42.75%).

**Figure 4:**
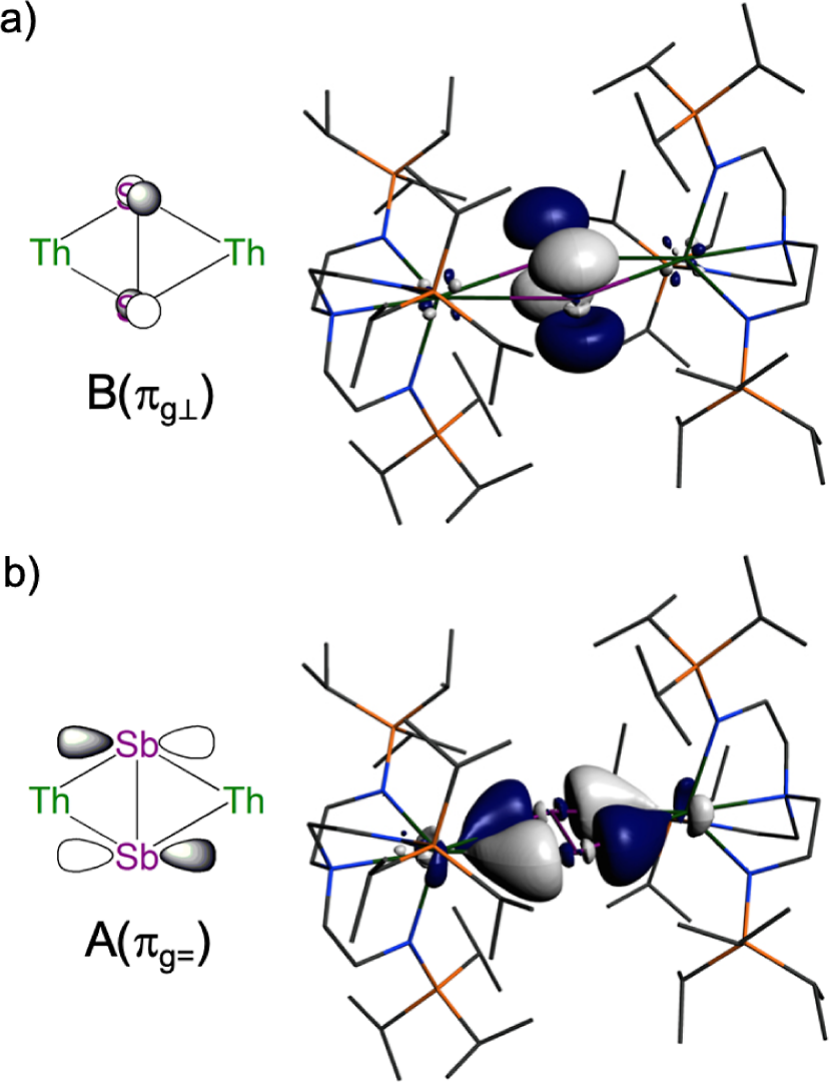
Selected frontier molecular orbital combinations of the anion component of **4** (**4′**). (a) B(π_g⊥_) π*-orbital symmetry combination for a *C*_2_-symmetric Th_2_Sb_2_ unit and the corresponding α-spin HOMO of **4′**. (b) A(π_g=_) π*-symmetry combination for a *C*_2_-symmetric Th_2_Sb_2_ unit and α-spin HOMO–1 of **4′** (the β-spin HOMO–1 is very similar). Hydrogen atoms are omitted for clarity.

The UV/vis/NIR spectrum of **4**, Figures 3a and S10, reveals four broad features centered at 10,718 (ε_max_ = 49 M^–1^ cm^–1^), 15,674 (ε_max_ = 2030 M^–1^ cm^–1^), 20,661 (ε_max_ = 1595 M^–1^ cm^–1^), and 23,981 (ε_max_ = 6388 M^–1^ cm^–1^) cm^–1^. TD-DFT calculations on **4′** (Table S3) reveal that these principally correspond to B(π_g⊥_) π*-f, B(π_g⊥_) π*-f, B(π_g⊥_) π*-f/d, and A(π_g=_) π*-f transitions, and hence, the first three involve the radical unpaired electron and the latter the doubly occupied in-plane Th–Sb π-bond; Figure 4 illustrates the B(π_g⊥_) and A(π_g=_) molecular orbitals. The first absorption corresponds to the HOMO–LUMO transition. This is a significantly different spectrum to that of **3a**,^57^Figure 3a, which does not exhibit any absorption maxima below 21,000 cm^–1^. Hence, the presence of the radical in **4** is revealed by the additional low energy absorptions and also the fact that the A(π_g=_) π*-f transitions are red-shifted by ∼2400 cm^–1^ for **4** compared to **3a**.

Confirmation of the presence of the paramagnetic Sb_2_^•3–^ radical trianion in **4** is afforded by SQUID magnetometry and EPR spectroscopy, Figures 3b–d and S11–S13. The effective magnetic moment of **4** at 300 K is 1.67 μ_B_ (0.35 cm^3^ mol^–1^ K), and this remains almost unchanged upon lowering the temperature (1.61 μ_B_, 0.33 cm^3^ mol^–1^ K, at 1.8 K), Figure 3b. This is consistent with the presence of a single unpaired electron in **4**,^74^ noting that the hypothetical spin only magnetic moment for a single unpaired electron is 1.73 μ_B_ (0.375 cm^3^ mol^–1^ K, for *g* = 2). At 1.8 K, magnetization versus field data for **4** saturate by 7 T (0.82 N_A_μ_B_), also supporting the presence of a single unpaired electron in **4**, Figure 3c. Neither [Li(DME)_3_][RSbSbR] (R = C_6_H_2_-2,6-(CH(SiMe_3_)_2_)_2_-4-C(SiMe_3_)_3_)^48^ nor [K(B18C6)(DME)][{HC(CMeNAr)_2_}Ga(NMe_2_)Sb]_2_^71^ have any reported magnetic data for comparison, but we note that the magnetism of **4** appears to be little affected by the heavy thorium ions.

The c.w. X-band EPR spectrum of polycrystalline **4** at 5 K, Figure 3d, reveals a well resolved spectrum with complex hyperfine structure due to the presence of ^121/123^Sb isotopes (*I* = 5/2, 57.25%; 7/2, 42.75%). Simulation suggests *g*_*x*_ = 2.29, *g*_*y*_ = 2.26, and *g*_*z*_ = 1.97 (*g*_iso_ = 2.18) with corresponding *A*(^121/123^Sb) of 116, 162, and 538 MHz. The *g*-values predict μ_eff_ = 1/2[(*g*_*x*_^2^ + *g*_*y*_^2^ + *g*_*z*_^2^)^1/2^] = 1.88 μ_B_, which is in fair agreement with the magnetic data. Moreover, DFT calculations on **4′** predict *g* values of *g*_*x*_ = 2.30, *g*_*y*_ = 2.22, and *g*_*z*_ = 1.98 (*g*_iso_ = 2.17), in excellent agreement with the experiment. The EPR data for **4** compare to those reported for [Li(DME)_3_][RSbSbR] (*g*_*x*_ = 1.961, *g*_*y*_ = 2.030, *g*_*z*_ = 2.368, *g*_iso_ = 2.13, *A* = 644, 350, 126 MHz)^49^ and [K(B18C6)(DME)][{HC(CMeNAr)_2_}Ga(NMe_2_)Sb]_2_ (*g*_*x*_ = 2.000, *g*_*y*_ = 2.051, *g*_*z*_ = 2.401, *g*_iso_ = 2.15, *A* = 120, 200, 560 MHz).^71^ The change in *g*-value ordering reflects the side-on coordination of the Sb_2_ unit in **4** compared to the “end-on” zigzag motif of the latter two compounds, and the data for **4** are distinct to single Sb-atom radicals and do not evidence any Th(III) character.^74−78^

### Density Functional Theory Calculations

As noted earlier, DFT calculations on **4′** reproduce key features of the IR, Raman, UV/vis/NIR, and EPR spectroscopic data, so they can be considered to provide a quantified electronic structure description of the anion of **4**.^72^

The computed multipole derived charge analysis reveals Th/Sb (av.) MDC_q_ charges for **3a** and **4′** of 2.29/–0.33 and 1.87/–0.44, respectively. Hence, on moving from Sb_2_^2–^ to Sb_2_^•3–^, the Th charge reduces reflecting more charge donation to Th, and the Sb_2_ unit charge becomes more negative, from −0.66 to −0.88 reflecting the additional electron in the latter. Inspection of the MDC_m_ spin density data for **4′** reveals only a modest net donation of electron density to Th (0.03 e), but the presence of the Sb_3_^•3–^ radical is supported by a net spin density of 0.94 e on the Sb_2_ unit. The Nalewajski–Mrozek Th–Sb (av.)/Sb–Sb bond indices for **3a** and **4′** are 0.50/2.02 and 0.62/1.44, which again supports the notion of Sb_3_^•3–^ being a superior donor than Sb_2_^2–^ and reduction of the formal Sb=Sb double bond character on moving from Sb_2_^2–^ to Sb_2_^•3–^ due to the presence of an additional electron in the π* manifold of the latter. For comparison, the analogous Th–N_amide_ and Th–N_amine_ bond indices are consistently ∼1.00 and ∼0.30 for both complexes.

The α-spin HOMO of **4′**, Figure 4a, which is the LUMO of **3a**, is the singly occupied B(π_g⊥_) π* orbital of the Sb_2_^•3–^ unit, and this is composed of 89% Sb (5p) and 11% Th (6d:5f 1:2) character, and hence, it is overwhelmingly of Sb_2_^•3–^ character with little Th character. The HOMO–1 of **4′**, Figure 4b, represents the corresponding principal Th–Sb interaction, and this is an A(π_g=_) in-plane π-character combination composed of 32% Th (6d) and 68% Sb (5p) character. In **3a**, this orbital is 22% Th (3:1 6d:5f) and 78% Sb (5p) character, and the increased Th character in **4′** is in-line with the above data suggesting that the more electron-rich Sb_2_^•3–^ is a better donor to Th than Sb_2_^2–^.

QTAIM analysis of the Sb–Sb and Th–Sb 3,–1 bond critical points (BCPs) in **3a** and **4′** reveal ρ/∇^2^ρ/*H* (energy)/ε (ellipticity) values of 0.06/0.03/–0.03/0.08 and 0.03/0.03/–0.05/0.15 (×4) for **3a** and 0.06/0.03/–0.02/0.15 and 0.03/0.03/–0.05/0.09 (×4) for **4′**. These data reflect rather polar bonding between such large element centers, slightly weaker Sb–Sb bonding on moving from Sb_2_^2–^ to Sb_2_^•3–^, as evidenced by the small reduction in *H* term on moving from **3a** to **4′**, and also that addition of the extra unpaired electron is resulting in a slightly less symmetrical charge distribution around the Sb–Sb internuclear axis. However, the changes to the BCP data are small, likely reflecting that the electronic structure changes principally occur in diffuse 5p-orbitals.

## Conclusions

In conclusion, sequestering of K^+^ during reduction affords a Th-stabilized Sb_2_^•3–^ radical trianion that is distinct from prior Sb_2_-derived radicals and that is unprecedented for d-/f-block metals, being the heaviest reported actinide-N_2_ radical analogue. SQUID magnetometry and EPR spectroscopy suggest that the Sb_2_^•3–^ radical trianion in **4** is reasonably well isolated due to its electrostatic binding to Th. The computational analysis reproduces key spectroscopic characterization data, and the resulting bonding analysis demonstrates the stronger donating capability of the Sb_2_^•3–^ radical trianion, and weaker Sb–Sb bond, than the Sb_2_^2–^ dianion form.

## Data Availability

All other data are provided in the Supporting Information or are available from the authors on reasonable request.

## Supporting Information Available

Experimental and computational details, X-ray crystallographic analysis of **4** and **5**, NMR spectra of **4**, experimental and computed infrared spectra of **4**, Raman spectrum of **4**, UV/vis/NIR spectrum of **4**, EPR spectrum of powdered **4**, SQUID magnetometry data on powdered **4**, XRD data for **4** and **5**, computational data for **4**, TD-DFT absorptions and optical modelling of **4** (PDF)

## References

[ref1] Winterbourn C. C. Reconciling the chemistry and biology of reactive oxygen species. Nat. Chem. Biol. 2008, 4, 278–286. 10.1038/nchembio.85. 18421291

[ref2] Tidwell T. Sunlight and free radicals. Nat. Chem. 2013, 5, 637–639. 10.1038/nchem.1703. 23881483

[ref3] Yan M.; Lo J. C.; Edwards J. T.; Baran P. S. Radicals: Reactive Intermediates with Translational Potential. J. Am. Chem. Soc. 2016, 138, 12692–12714. 10.1021/jacs.6b08856. 27631602 PMC5054485

[ref4] Studer A.; Curran D. P. Catalysis of Radical Reactions: A Radical Chemistry Perspective. Angew. Chem., Int. Ed. 2016, 55, 58–102. 10.1002/anie.201505090. 26459814

[ref5] Leifert D.; Studer A. The Persistent Radical Effect in Organic Synthesis. Angew. Chem., Int. Ed. 2020, 59, 74–108. 10.1002/anie.201903726. 31116479

[ref6] Stubbe J.; Nocera D. G. Radicals in Biology: Your Life Is in Their Hands. J. Am. Chem. Soc. 2021, 143, 13463–13472. 10.1021/jacs.1c05952. 34423635 PMC8735831

[ref7] Roussel P.; Scott P. Complex of Dinitrogen with Trivalent Uranium. J. Am. Chem. Soc. 1998, 120, 1070–1071. 10.1021/ja972933+.

[ref8] Chiesa M.; Giamello E.; Murphy D. M.; Pacchioni G.; Paganini M. C.; Soave R.; Sojka Z. Reductive Activation of the Nitrogen Molecule at the Surface of “Electron-Rich” MgO and CaO. The N_2_^–^ Surface Adsorbed Radical Ion. J. Phys. Chem. B 2001, 105, 497–505. 10.1021/jp002794+.

[ref9] Evans W. J.; Ulibarri T. A.; Ziller J. W. Isolation and X-ray crystal structure of the first dinitrogen complex of an f-element metal, [(C_5_Me_5_)_2_Sm]_2_N_2_. J. Am. Chem. Soc. 1988, 110, 6877–6879. 10.1021/ja00228a043.

[ref10] Evans W. J.; Fang M.; Zucchi G.; Furche F.; Ziller J. W.; Hoekstra R. M.; Zink J. I. Isolation of Dysprosium and Yttrium Complexes of a Three-Electron Reduction Product in the Activation of Dinitrogen, the (N_2_)^3–^ Radical. J. Am. Chem. Soc. 2009, 131, 11195–11202. 10.1021/ja9036753. 19610635

[ref11] Falcone M.; Chatelain L.; Scopelliti R.; Živković I.; Mazzanti M. Nitrogen reduction and functionalization by a multimetallic uranium nitride complex. Nature 2017, 547, 332–335. 10.1038/nature23279. 28726827

[ref12] Falcone M.; Barluzzi L.; Andrez J.; Fadaei Tirani F.; Zivkovic I.; Fabrizio A.; Corminboeuf C.; Severin K.; Mazzanti M. The role of bridging ligands in dinitrogen reduction and functionalization by uranium multimetallic complexes. Nat. Chem. 2019, 11, 154–160. 10.1038/s41557-018-0167-8. 30420774

[ref13] Rinehart J. D.; Fang M.; Evans W. J.; Long J. R. Strong exchange and magnetic blocking in N_2_^3–^-radical-bridged lanthanide complexes. Nat. Chem. 2011, 3, 538–542. 10.1038/nchem.1063. 21697874

[ref14] Rinehart J. D.; Fang M.; Evans W. J.; Long J. R. A N_2_^3–^-Radical-Bridged Terbium Complex Exhibiting Magnetic Hysteresis at 14 K. J. Am. Chem. Soc. 2011, 133, 14236–14239. 10.1021/ja206286h. 21838285

[ref15] Demir S.; Gonzalez M. I.; Darago L. E.; Evans W. J.; Long J. R. Giant coercivity and high magnetic blocking temperatures for N_2_^3–^ radical-bridged dilanthanide complexes upon ligand dissociation. Nat. Commun. 2017, 8, 2144 10.1038/s41467-017-01553-w. 29247236 PMC5732206

[ref16] Korobkov I.; Gambarotta S.; Yap G. P. A. A Highly Reactive Uranium Complex Supported by the Calix[4]tetrapyrrole Tetraanion Affording Dinitrogen Cleavage, Solvent Deoxygenation, and Polysilanol Depolymerization. Angew. Chem., Int. Ed. 2002, 41, 3433–3436. 10.1002/1521-3773(20020916)41:18<3433::aid-anie3433>3.0.co;2-v. 12298055

[ref17] Xin X.; Douair I.; Zhao Y.; Wang S.; Maron L.; Zhu C. Dinitrogen cleavage by a heterometallic cluster featuring multiple uranium–rhodium bonds. J. Am. Chem. Soc. 2020, 142, 15004–15011. 10.1021/jacs.0c05788. 32786768

[ref18] Xin X.; Douair I.; Zhao Y.; Wang S.; Maron L.; Zhu C. Dinitrogen cleavage and hydrogenation to ammonia with a uranium complex. Natl. Sci. Rev. 2023, 10, nwac144 10.1093/nsr/nwac144. 36950222 PMC10026940

[ref19] Du J.; Cobb P. J.; Ding J.; Mills D. P.; Liddle S. T. f-Element heavy pnictogen chemistry. Chem. Sci. 2024, 15, 13–45. 10.1039/D3SC05056D. PMC10732230 38131077

[ref20] Fermín M. C.; Ho J.; Stephan D. W. Substituent-Free P_1_, P_2_, and P_3_ Complexes of Zirconium. J. Am. Chem. Soc. 1994, 116, 6033–6034. 10.1021/ja00092a090.

[ref21] Figueroa J. S.; Cummins C. C. The Niobaziridine-Hydride Functional Group: Synthesis and Divergent Reactivity. J. Am. Chem. Soc. 2003, 125, 4020–4021. 10.1021/ja028446y. 12670202

[ref22] Figueroa J. S.; Cummins C. C. Diorganophosphanylphosphinidenes as Complexed Ligands: Synthesis via an Anionic Terminal Phosphide of Niobium. Angew. Chem., Int. Ed. 2004, 43, 984 10.1002/anie.200352779. 14966886

[ref23] Piro N. A.; Figueroa J. S.; McKellar J. T.; Cummins C. C. Triple-Bond Reactivity of Diphosphorus Molecules. Science 2006, 313, 1276–1279. 10.1126/science.1129630. 16946068

[ref24] Piro N. A.; Cummins C. C. P_2_ Addition to Terminal Phosphide M≡P Triple Bonds: A Rational Synthesis of cyclo-P_3_ Complexes. J. Am. Chem. Soc. 2008, 130, 9524–9535. 10.1021/ja802080m. 18588294

[ref25] Hulley E. B.; Wolczanski P. T.; Lobkovsky E. B. [(silox)_3_M]_2_(μ:η^1^,η^1^-P_2_) (M = Nb, Ta) and [(silox)_3_Nb]_2_{μ:η^2^,η^2^-P_2_ (^c^P_3_–^c^P_3_)} from (silox)_3_M (M = NbPMe_3_, Ta) and P_4_ (silox = ^*t*^Bu_3_SiO). Chem. Commun. 2009, 6412–6414. 10.1039/b911275h. 19841793

[ref26] Yao S.; Szilvási T.; Lindenmaier N.; Xiong Y.; Inoue S.; Adelhardt M.; Sutter J.; Meyer K.; Driess M. Reductive cleavage of P_4_ by iron(I) centres: synthesis and structural characterisation of Fe_2_(P_2_)_2_ complexes with two bridging P_2_^2-^ ligands. Chem. Commun. 2015, 51, 6153–6156. 10.1039/C5CC00147A. 25747898

[ref27] Liu L.; Ruiz D. A.; Dahcheh F.; Bertrand G.; Suter R.; Tondreau A. M.; Grützmacher H. Isolation of Au-, Co-η^1^PCO and Cu-η^2^PCO complexes, conversion of an Ir-η^1^PCO complex into a dimetalladiphosphene, and an interaction-free PCO anion. Chem. Sci. 2016, 7, 2335–2341. 10.1039/C5SC04504E. 29997776 PMC6003603

[ref28] Xiong Y.; Yao S.; Szilvási T.; Ballestero-Martínez E.; Grützmacher H.; Driess M. Unexpected Photodegradation of a Phosphaketenyl-Substituted Germyliumylidene Borate Complex. Angew. Chem., Int. Ed. 2017, 56, 4333–4336. 10.1002/anie.201701337. 28295977

[ref29] Grant L. N.; Pinter B.; Manor B. C.; Suter R.; Grützmacher H.; Mindiola D. J. A Planar Ti_2_P_2_ Core Assembled by Reductive Decarbonylation of ^–^O-C≡P and P-P Radical Coupling. Chem.—Eur. J. 2017, 23, 6272–6276. 10.1002/chem.201701054. 28297126

[ref30] Hierlmeier G.; Hinz A.; Wolf R.; Goicoechea J. M. Synthesis and Reactivity of Nickel-Stabilised μ^2^:η^2^,η^2^-P_2_, As_2_ and PAs Units. Angew. Chem., Int. Ed. 2018, 57, 431–436. 10.1002/anie.201710582. 29152826

[ref31] Abbenseth J.; Diefenbach M.; Hinz A.; Alig L.; Würtele C.; Goicoechea J. M.; Holthausen M. C.; Schneider S. Oxidative Coupling of Terminal Rhenium Pnictide Complexes. Angew. Chem., Int. Ed. 2019, 58, 10966–10970. 10.1002/anie.201905130. 31179626

[ref32] Wang Y.; Szilvasi T.; Yao S.; Driess M. A bis(silylene)-stabilized diphosphorus compound and its reactivity as a monophosphorus anion transfer reagent. Nat. Chem. 2020, 12, 801–807. 10.1038/s41557-020-0518-0. 32807885

[ref33] Du J.; Hunger D.; Seed J. A.; Cryer J. D.; King D. M.; Wooles A. J.; van Slageren J.; Liddle S. T. Dipnictogen f-Element Chemistry: A Diphosphorus Uranium Complex. J. Am. Chem. Soc. 2021, 143, 5343–5348. 10.1021/jacs.1c02482. 33792307

[ref34] Sun J.; Verplancke H.; Schweizer J. I.; Diefenbach M.; Würtele C.; Otte M.; Tkach I.; Herwig C.; Limberg C.; Demeshko S.; Holthausen M. C.; Schneider S. Stabilizing P≡P: P_2_^2–^, P_2_^•–^, and P_2_^0^ as bridging ligands. Chem 2021, 7, 1952–1962. 10.1016/j.chempr.2021.06.006.

[ref35] Wang S.; Sears J. D.; Moore C. E.; Rheingold A. L.; Neidig M. L.; Figueroa J. S. Side-on coordination of diphosphorus to a mononuclear iron center. Science 2022, 375, 1393–1397. 10.1126/science.abn7100. 35324298 PMC9210196

[ref36] Du J.; Atkinson B. E.; Seed J. A.; Sheppard R. F.; Tuna F.; Wooles A. J.; Chilton N. F.; Liddle S. T. Strong Uranium-Phosphorus Antiferromagnetic Exchange Coupling in a Crystalline Diphosphorus Radical Trianion Actinide Complex. Chem 2024, 102337 10.1016/j.chempr.2024.10.004.

[ref37] Sullivan P. J.; Rheingold A. L. Group VIB complexes containing arsenic-arsenic double bonds. Synthesis and crystallographic characterization of [η^5^-C_5_H_5_M(CO)_2_]_2_(mu.-eta.2-As_2_) (M = Mo or W). Organometallics 1982, 1, 1547–1549. 10.1021/om00071a032.

[ref38] Huttner G.; Sigwarth B.; Scheidsteger O.; Zsolnai L.; Orama O. Diarsenic, As_2_, as a 4-, 6-, or 8-electron donor ligand. Organometallics 1985, 4, 326–332. 10.1021/om00121a023.

[ref39] Adams K. V.; Choi N.; Conole G.; Davies J. E.; King J. D.; Mays M. J.; McPartlin M.; Raithby P. R. Synthesis, reactivity and structural characterization of some heterometallic complexes containing naked arsenic ligands. J. Chem. Soc., Dalton Trans. 1999, 3679–3686. 10.1039/a905688b.

[ref40] Abraham M. Y.; Wang Y.; Xie Y.; Gilliard R. J.; Wei P.; Vaccaro B. J.; Johnson M. K.; Schaefer H. F.; Schleyer P. v. R.; Robinson G. H. Oxidation of Carbene-Stabilized Diarsenic: Diarsene Dications and Diarsenic Radical Cations. J. Am. Chem. Soc. 2013, 135, 2486–2488. 10.1021/ja400219d. 23363453

[ref41] Gardner B. M.; Balázs G.; Scheer M.; Wooles A. J.; Tuna F.; McInnes E. J. L.; McMaster J.; Lewis W.; Blake A. J.; Liddle S. T. Isolation of Elusive HAsAsH in a Crystalline Diuranium(IV) Complex. Angew. Chem., Int. Ed. 2015, 54, 15250–15254. 10.1002/anie.201508600. PMC4691330 26510123

[ref42] Graßl C.; Bodensteiner M.; Zabel M.; Scheer M. Synthesis of arsenic-rich Asn ligand complexes from yellow arsenic. Chem. Sci. 2015, 6, 1379–1382. 10.1039/C4SC03543G. 29560225 PMC5811125

[ref43] Tuscher L.; Helling C.; Wölper C.; Frank W.; Nizovtsev A. S.; Schulz S. A General Route to Metal-Substituted Dipnictenes of the Type [L(X)M]_2_E_2_. Chem.—Eur. J. 2018, 24, 3241–3250. 10.1002/chem.201705233. 29266416

[ref44] Ballestero-Martínez E.; Szilvási T.; Hadlington T. J.; Driess M. From As-Zincoarsasilene (LZn-As = SiL′) to Arsaethynolato (As≡C–O) and Arsaketenylido (O = C=As) Zinc Complexes. Angew. Chem., Int. Ed. 2019, 58, 3382–3386. 10.1002/anie.201813521. 30620428

[ref45] Sharma M. K.; Blomeyer S.; Neumann B.; Stammler H.-G.; van GastelHinz M. A.; Ghadwal R. S.-D. A.; Ghadwal R. S. Crystalline Divinyldiarsene Radical Cations and Dications. Angew. Chem., Int. Ed. 2019, 58, 17599–17603. 10.1002/anie.201909144. PMC6899687 31553520

[ref46] Schoo C.; Bestgen S.; Egeberg A.; Seibert J.; Konchenko S. N.; Feldmann C.; Roesky P. W. Samarium Polyarsenides Derived from Nanoscale Arsenic. Angew. Chem., Int. Ed. 2019, 58, 4386–4389. 10.1002/anie.201813370. 30614173

[ref47] Reinfandt N.; Schoo C.; Dütsch L.; Köppe R.; Konchenko S. N.; Scheer M.; Roesky P. W. Synthesis of Unprecedented 4d/4f-Polypnictogens. Chem.—Eur. J. 2021, 27, 3974–3978. 10.1002/chem.202003905. 33010187 PMC7986065

[ref48] Sieg G.; Fischer M.; Dankert F.; Siewert J.-E.; Hering-Junghans C.; Werncke G. A diarsene radical anion. Chem. Commun. 2022, 58, 9786–9789. 10.1039/D2CC03237F. 35971739

[ref49] Sasamori T.; Mieda E.; Nagahora N.; Sato K.; Shiomi D.; Takui T.; Hosoi Y.; Furukawa Y.; Takagi N.; Nagase S.; Tokitoh N. One-Electron Reduction of Kinetically Stabilized Dipnictenes: Synthesis of Dipnictene Anion Radicals. J. Am. Chem. Soc. 2006, 128, 12582–12588. 10.1021/ja064062m. 16984209

[ref50] Sakagami M.; Sasamori T.; Sakai H.; Furukawa Y.; Tokitoh N. 1,2-Bis(ferrocenyl)-Substituted Distibene and Dibismuthene: Sb=Sb and Bi=Bi Units as π Spacers between Two Ferrocenyl Units. Chem.—Asian J. 2013, 8, 690–693. 10.1002/asia.201201227. 23377927

[ref51] Kretschmer R.; Ruiz D. A.; Moore C. E.; Rheingold A. L.; Bertrand G. One-, Two-, and Three-Electron Reduction of a Cyclic Alkyl(amino)carbene–SbCl_3_ Adduct. Angew. Chem., Int. Ed. 2014, 53, 8176–8179. 10.1002/anie.201404849. 24961494

[ref52] Krüger J.; Schoening J.; Ganesamoorthy C.; John L.; Wölper C.; Schulz S. Synthesis and X-ray Crystal Structures of Ga-substituted Distibenes [L(X)GaSb]_2_. Z. Anorg. Allg. Chem. 2018, 644, 1028–1033. 10.1002/zaac.201800204.

[ref53] Schoo C.; Bestgen S.; Egeberg A.; Klementyeva S.; Feldmann C.; Konchenko S. N.; Roesky P. W. Samarium Polystibides Derived from Highly Activated Nanoscale Antimony. Angew. Chem., Int. Ed. 2018, 57, 5912–5916. 10.1002/anie.201802250. 29528543

[ref54] Song L.; Schoening J.; Wölper C.; Schulz S.; Schreiner P. R. Role of London Dispersion Interactions in Ga-Substituted Dipnictenes. Organometallics 2019, 38, 1640–1647. 10.1021/acs.organomet.9b00072.

[ref55] Weinert H. M.; Wölper C.; Schulz S. Synthesis of distibiranes and azadistibiranes by cycloaddition reactions of distibenes with diazomethanes and azides. Chem. Sci. 2022, 13, 3775–3786. 10.1039/d2sc00314g. 35432897 PMC8966720

[ref56] Weinert H. M.; Schulte Y.; Gehlhaar A.; Wölper C.; Haberhauer G.; Schulz S. Metal-coordinated distibene and dibismuthene dications-isoelectronic analogues of butadiene dications. Chem. Commun. 2023, 59, 7755–7758. 10.1039/D3CC01844J. 37272311

[ref57] Du J.; Dollberg K.; Seed J. A.; Wooles A. J.; von Hänisch C.; Liddle S. T. Thorium(IV)–antimony complexes exhibiting single, double, and triple polar covalent metal–metal bonds. Nat. Chem. 2024, 16, 780–790. 10.1038/s41557-024-01448-6. 38378948

[ref58] Xu L.; Bobev S.; El-Bahraoui J.; Sevov S. C. A Naked Diatomic Molecule of Bismuth, [Bi_2_]^2-^, with a Short Bi–Bi Bond: Synthesis and Structure. J. Am. Chem. Soc. 2000, 122, 1838–1839. 10.1021/ja992422i.

[ref59] Evans W. J.; Gonzales S. L.; Ziller J. W. Organosamarium-Mediated Synthesis of Bismuth Bismuth Bonds: X-ray Crystal Structure of the First Dibismuth Complex Containing a Planar M_2_(μ-η^2^:η^2^-Bi_2_) Unit. J. Am. Chem. Soc. 1991, 113, 9880–9882. 10.1021/ja00026a040.

[ref60] Prabusankar G.; Gemel C.; Parameswaran P.; Flener C.; Frenking G.; Fischer R. A. A Short Bi = Bi Bond Supported by a Metalloid Group 13 Ligand. Angew. Chem., Int. Ed. 2009, 48, 5526–5529. 10.1002/anie.200902172. 19554586

[ref61] Zhang P.; Nabi R.; Staab J. K.; Chilton N. F.; Demir S. Taming Super-Reduced Bi_2_^3–^ Radicals with Rare Earth Cations. J. Am. Chem. Soc. 2023, 145, 9152–9163. 10.1021/jacs.3c01058. 37043770 PMC10141245

[ref62] Miyake K. Formation of Cesium Antimonide. I. Electrical Resistance of the Film of Cesium-Antimony System. J. Appl. Phys. 1960, 31, 76–81. 10.1063/1.1735422.

[ref63] Gascoin F.; Sevov S. C. Synthesis and Characterization of A_3_Bi_2_ (A = K, Rb, Cs) with Isolated Diatomic Dianion of Bismuth, [Bi_2_]^2–^, and an Extra Delocalized Electron. J. Am. Chem. Soc. 2000, 122, 10251–10252. 10.1021/ja002606t.

[ref64] Lichtenberger N.; Wilson R. J.; Eulenstein A. R.; Massa W.; Clerac R.; Weigend F.; Dehnen S. Main Group Metal–Actinide Magnetic Coupling and Structural Response Upon U^4+^ Inclusion Into Bi, Tl/Bi, or Pb/Bi Cages. J. Am. Chem. Soc. 2016, 138, 9033–9036. 10.1021/jacs.6b04363. 27392253

[ref65] Rookes T. M.; Wildman E. P.; Balázs G.; Gardner B. M.; Wooles A. J.; Gregson M.; Tuna F.; Scheer M.; Liddle S. T. Actinide-Pnictide (An-Pn) Bonds Spanning Non-Metal, Metalloid, and Metal Combinations (An = U, Th; Pn = P, As, Sb, Bi). Angew. Chem., Int. Ed. 2018, 57, 1332–1336. 10.1002/anie.201711824. PMC5814731 29232498

[ref66] Eulenstein A. R.; Franzke Y. J.; Lichtenberger N.; Wilson R. J.; DeubnerKraus H. F.; Clérac R.; Weigend F.; Dehnen S.; Dehnen S. Substantial π-aromaticity in the anionic heavy-metal cluster [Th@Bi_12_]^4–^. Nat. Chem. 2021, 13, 149–155. 10.1038/s41557-020-00592-z. 33288891

[ref67] Rookes T. M.; Balázs G.; Gardner B. M.; Wooles A. J.; Scheer M.; Liddle S. T. Actinide-Pnictide Chemistry: A Uranium Primary Alkyl Stibinide and a Diuranium Hexaantimonide-Tetralithium Zintl Cluster. ChemistryEurope 2023, 1, e202300067 10.1002/ceur.202300067.

[ref68] Du J.; Dollberg K.; Seed J. A.; Wooles A. J.; Von Hänisch C.; Liddle S. T. f-Element Zintl Chemistry: Actinide-Mediated Dehydrocoupling of H_2_Sb^1–^ Affords the Tri-Thorium and -Uranium Undeca-Antimontriide Zintl Clusters [{An(Tren^TIPS^)}_3_(μ_3_-Sb_11_)] (An = Th, U; Tren^TIPS^ = {N(CH_2_CH_2_NSi^i^Pr_3_)_3_}^3–^). Inorg. Chem. 2024, 63, 20153–20160. 10.1021/acs.inorgchem.4c00923. 38767623 PMC11523227

[ref69] Dollberg K.; Schneider S.; Richter R.-M.; Dunaj T.; von Hänisch C. Synthesis and Application of Alkali Metal Antimonide-A New Approach to Antimony Chemistry. Angew. Chem., Int. Ed. 2022, 61, e202213098 10.1002/anie.202213098. PMC10099276 36301563

[ref70] Wildman E. P.; Balázs G.; Wooles A. J.; Scheer M.; Liddle S. T. Thorium-Phosphorus Triamidoamine Complexes Containing Th-P Single- and Multiple-Bond Interactions. Nat. Commun. 2016, 7, 12884 10.1038/ncomms12884. 27682617 PMC5056418

[ref71] Weinert H. M.; Wolper C.; Haak J.; Cutsail G. E.; Schulz S. Synthesis, structure and bonding nature of heavy dipnictene radical anions. Chem. Sci. 2021, 12, 14024–14032. 10.1039/D1SC04230K. 34760185 PMC8565390

[ref72] See Supporting Information for full experimental and computational details.

[ref73] Pyykkö P. Additive Covalent Radii for Single-, Double-, and Triple-Bonded Molecules and Tetrahedrally Bonded Crystals: A Summary. J. Phys. Chem. A 2015, 119, 2326–2337. 10.1021/jp5065819. 25162610

[ref74] Ganesamoorthy C.; Helling C.; Wölper C.; Frank W.; Bill E.; Cutsail G. E. III; Schulz S. From stable Sb- and Bi-centered radicals to a compound with a Ga = Sb double bond. Nat. Commun. 2018, 9, 87 10.1038/s41467-017-02581-2. 29311607 PMC5758792

[ref75] Kretschmer R.; Ruiz D. A.; Moore C. E.; Rheingold A. L.; Bertrand G. One-, Two-, and Three-Electron Reduction of a Cyclic Alkyl(amino)carbene-SbCl_3_ Adduct. Angew. Chem., Int. Ed. 2014, 53, 8176–8179. 10.1002/anie.201404849. 24961494

[ref76] Ishida S.; Hirakawa F.; Furukawa K.; Yoza K.; Iwamoto T. Persistent Antimony- and Bismuth-Centred Radicals in Solution. Angew. Chem., Int. Ed. 2014, 53, 11172–11176. 10.1002/anie.201405509. 25066471

[ref77] Li T.; Wei H.; Fang Y.; Wang L.; Chen S.; Zhang Z.; Zhao Y.; Tan G.; Wang X. Elusive Antimony-Centered Radical Cations: Isolation, Characterization, Crystal Structures, and Reactivity Studies. Angew. Chem., Int. Ed. 2017, 56, 632–636. 10.1002/anie.201610334. 27930850

[ref78] Nguyen J. Q.; Anderson-Sanchez L. M.; Moore W. N. G.; Ziller J. W.; Furche F.; Evans W. J. Replacing Trimethylsilyl with Triisopropylsilyl Provides Crystalline (C_5_H_4_SiR_3_)_3_Th Complexes of Th(III) and Th(II). Organometallics 2023, 42, 2927–2937. 10.1021/acs.organomet.3c00343.

