# Supplementary material for: Dipnictogen Radical Chemistry: A Dithorium-Supported Distibene Radical Trianion
Source: J Am Chem Soc. 2025 Jan 23;147(5):4430–7. doi: 10.1021/jacs.4c15431 (PMC11803619; doi:10.1021/jacs.4c15431)
Supplement: Supplementary file 1 — ja4c15431_si_001.pdf [file ja4c15431_si_001.pdf]

– *Supporting Information* –

Dipnictogen Radical Chemistry: A Dithorium Supported Distibene Radical Trianion

Jingzhen Du,[1,2] Kevin Dollberg,[3] John A. Seed,[1] Floriana Tuna,[4] Ashley J. Wooles,[1] Carsten von Hänisch,[3*] Stephen T. Liddle[1*]

[1] Department of Chemistry and Centre for Radiochemistry Research, The University of Manchester, Oxford Road, Manchester, M13 9PL, UK.

[2] Present address: College of Chemistry, Zhengzhou University, Zhengzhou, 450001, China.

[3] Fachbereich Chemie, Philipps-Universität Marburg, Hans-Meerwein-Straße 4, 35043 Marburg, Germany.

[4] Department of Chemistry and Photon Science Institute, The University of Manchester, Oxford Road, Manchester, M13 9PL, UK.

*Email: haenisch@chemie.uni-marburg.de; steve.liddle@manchester.ac.uk

METHODS

Experimental

General Considerations

All manipulations were carried out using Schlenk techniques, or an MBraun UniLab glovebox, under an atmosphere of dry nitrogen. Solvents were dried by passage through activated alumina towers and

degassed before use. All solvents were stored over potassium mirrors except for ethers, which were stored over activated 4 Å sieves. Deuterated solvent was distilled from potassium, degassed by three freeze-pump-thaw cycles and stored under dinitrogen. 2.2.2-cryptand was dried under dynamic vacuum for 24 hr prior to use. KC_8 and the 77:23% mixture of $[\{Th(Tren^{TIPS})\}_2(\mu\text{-}\eta^2\text{:}\eta^2\text{-}Sb_2)]$ (**3a**):$[\{Th(Tren^{TIPS})\}_2(\mu\text{-}SbH)]$ (**3b**) were prepared as described previously.[1,2] Single crystals were examined on Rigaku XtaLAB Synergy-S diffractometer equipped with a HyPix 6000HE photon counting pixel array detector with mirror-monochromated CuKα (λ = 1.5418 Å) radiation. Intensities were integrated from a sphere of data recorded on narrow (0.38 or 0.5°) frames by ω rotation. Cell parameters were refined from the observed positions of all strong reflections in each data set. Gaussian grid face-indexed absorption corrections with a beam profile correction were applied. The structures were solved by dual methods using SHELXT,[3] and all non-hydrogen atoms were refined by full-matrix least-squares on all unique F^2 values with anisotropic displacement parameters; exceptions are noted in the respective cif files. Except where noted, hydrogen atoms were refined with constrained geometries and riding thermal parameters; U_{iso}(H) was set at 1.2 (1.5 for methyl groups) times U_{eq} of the parent atom. The largest features in final difference syntheses were close to heavy atoms and were of no chemical significance. CrysAlisPro was used for control and integration,[4] and SHELXL and Olex2 were employed for structure refinement.[5,6] ORTEP-3 and POV-Ray were employed for molecular graphics.[7,8] ^1H, $^{13}C\{^1H\}$, and $^{29}Si\{^1H\}$ NMR spectra were recorded on a Bruker 400 spectrometer operating at 400.1, 100.6, and 79.48 MHz, or on a JEOL JNM-ECZ 400 MHz spectrometer operating at 399.78, 100.52, and 79.42 MHz respectively; samples were prepared in a glovebox and placed in J. Young PTFE 5mm screw-topped borosilicate NMR tubes and chemical shifts are quoted in ppm and are relative to TMS (^1H, ^{13}C, and ^{29}Si). ATR-IR spectra were recorded on a Bruker Alpha spectrometer with a Platinum-ATR module in the glovebox. Raman spectra were recorded on a Horiba XploRA Plus Raman microscope using a 532 nm laser with a power of 1.5 mW. The power was adjusted using a power filter to inhibit sample decomposition. UV/vis/NIR spectra were recorded on a Perkin Elmer LAMBDA 750 spectrometer. Data were collected in a 1mm path-

length cuvette loaded in an MBraun glovebox and were run versus the appropriate solvent. EPR data were measured using an X-band (9.44 GHz) Bruker Elexsys E500 spectrometer equipped with an ER4118SMS5 resonator at 5 K. Powdered samples were loaded into 3.9 mm (OD) quartz tubes in a glovebox under argon or dinitrogen, and the tubes were flame sealed under vacuum using a MAPP-oxygen torch prior to experiments. Field corrections were applied to the raw data using Bruker strong pitch ($g = 2.0026$) as a reference. Variable-temperature magnetic moment data were recorded in an applied direct current (DC) field of 0.1 Tesla on a Quantum Design MPMS3 superconducting quantum interference device magnetometer using recrystallized powdered samples. Measurements were performed in dc scan mode using 40 mm scan length and 6 s scan time. Samples were carefully checked for purity and data reproducibility between independently prepared batches. Samples were crushed with a mortar and pestle under an argon atmosphere and immobilized in an eicosane matrix within 400 MHz Wilmad borosilicate NMR tubes to prevent sample reorientation during measurements. The tube was flame-sealed under dynamic vacuum (1×10^{-3} mbar) to a length of approximately 3 cm and mounted in the centre of a drinking straw, with the straw fixed to the end of an MPMS 3 sample rod. Care was taken to ensure complete thermalization of the sample before each data point was measured by employing delays at each temperature point as well as a slow cooling rate (2.5 K/min from 300 to 100 K; 1 K/min from 100 to 50 K; 0.5 K/min from 50 to 1.8 K). The sample was held at 1.8 K for 60 minutes before isothermal magnetization measurements to account for slow thermal equilibration of the sample. Diamagnetic corrections were applied using tabulated Pascal constants. Measurements were corrected for the effect of the blank sample holders (flame sealed Wilmad NMR tube and straw) and eicosane matrix. CHN microanalyses were carried out by Martin Jennings and Anne Davies at the University of Manchester.

Preparation of [K(2.2.2-cryptand)][{Th(Tren^{TIPS})}$_2$(μ-η^2:η^2-Sb$_2$)] (4)

Benzene (10 mL), was added to a 77:23% mixture of **3a**:**3b** (0.19 g, 0.10 mmol), KC_8 (0.02 g, 0.15 mmol), and 2.2.2-cryptand (0.04 g, 0.11 mmol) forming a green suspension. The mixture was stirred

for 24 hours with the exclusion of light, turning into a dark green suspension. Volatiles were removed *in vacuo* and the residue was extracted with THF (5 mL) and filtered to give an emerald green solution. The solution was concentrated to 2 mL and Et_2O (4 mL) was layered on top. Storage of the layered mixture at (–35 °C) for 24 hr afforded dark green crystals of **4**. The mother liquor was decanted away and the dark green crystals, which proved suitable for single crystal X-ray diffraction studies, were washed with cold Et_2O (2 × 2 mL) and dried *in vacuo*. Yield: 0.14 g, 61%. Crystals of **4** can also be obtained by diffusing pentane into a concentrated THF solution, however under those conditions a small amount of red crystals also co-crystallize. The red crystals were identified by single crystal X-ray diffraction studies to be $[K(2.2.2\text{-cryptand})]_3[\{Th(Tren^{TIPS})(\mu\text{-}\eta^3\text{:}\eta^3\text{-}Sb_3)\}_2(\mu\text{-}K)]$ (**5**). Due to the very small amount of this by-product (<1%) **5** was not characterized further. Anal. Calcd for $C_{84}H_{186}KN_{10}O_6Sb_2Si_6Th_2(Et_2O)$: C, 43.64; H, 8.16; N, 5.78%. Found: C, 43.92; H, 8.20; N, 5.61%. ^1H NMR (D_8-THF, 298 K): δ (ppm) 6.72 (br, 12H, NCH_2CH_2NSi), 3.83 (br, 18H, $CH(CH_3)_2$), 3.55 (m, 12H, $NCH_2CH_2OCH_2$), 3.33 (br, 108H, $CH(CH_3)_2$), 2.56 (s, 12H, $NCH_2CH_2OCH_2$), 1.78 (s, 12H, $NCH_2CH_2OCH_2$), −5.63 (br, 12H, NCH_2CH_2NSi). $^{13}C\{^1H\}$ NMR (D_8-THF, 298 K): δ (ppm) 75.67 ($NCH_2CH_2NSiPr^i_3$), 71.51 ($NCH_2CH_2OCH_2$), 68.65 ($NCH_2CH_2OCH_2$), 54.92 ($NCH_2CH_2OCH_2$), 26.56 ($NCH_2CH_2NSiPr^i_3$), 21.63 ($NCH_2CH_2NSiPr^i_3$), 16.87 ($NCH_2CH_2NSiPr^i_3$). $^{29}Si\{^1H\}$ NMR (D_8-THF, 298 K): δ (ppm) 0.08. ATR-IR ν/cm^{-1}: 2935 (m), 2881 (m), 2854 (s), 1458 (m), 1381 (w), 1355 (w), 1295 (w), 1276 (w), 1258 (w), 1104 (m), 1040 (m), 1013 (m), 934 (s), 880 (m), 794 (m), 736 (vs), 668 (s), 625 (m), 571 (w), 515 (w), 441 (w). UV/Vis/NIR (THF, 10mM, 298 K) Energy/cm^{-1} (ε/dm^3 mol^{-1} cm^{-1}): 10718 (49), 15674 (2030), 19120 (744), 20661 (1595), 21008 (1799), 21142 (1870), 21978 (2374), 22371 (2849), 22883 (3984), 23981 (6388), 24814 (5163), 26178 (5215), 26525 (7029), 27174 (20000). Raman (crystalline, 532 nm, 1.5 mW) ν/cm^{-1}: 2971, 2942, 2872, 2055, 1455, 1301, 221, 211, 202, 194, 187, 179, 173, 164, 158, 150, 137, 129, 125, 122, 114, 108, 104, 98, 93, 87, 79, 75, 71, 58, 50.

Computational

General

Calculations on **4** focussed on the anion portion of **4**, referred to as **4'**, and were performed using coordinates derived from the crystal structure as the starting point. No constraints were imposed on the structure during the geometry optimization. The calculations were performed using the Amsterdam Density Functional (ADF) suite version 2017 with standard convergence criteria.[9,10] The DFT geometry optimizations employed Slater type orbital (STO) triple-ζ-plus polarization all-electron basis sets (from the Dirac and ZORA/TZP database of the ADF suite). Scalar relativistic approaches (spin-orbit neglected) were used within the ZORA Hamiltonian[11-13] for the inclusion of relativistic effects and the local density approximation (LDA) with the correlation potential due to Vosko *et al* was used in all of the calculations.[14] Generalized gradient approximation (GGA) corrections were performed using the functionals of Becke and Perdew.[15,16] Analytical frequency calculations were carried out within the ADF program. TD-DFT calculations were carried out within the ADF program using the SAOP functional on the geometry optimized coordinates in a solvent continuum (COSMO model, THF). EPR *g*-value spin orbit calculations were carried out within the ADF program using the PBE0 functional (25% Hartree-Fock exchange) on the geometry optimized coordinates. Scalar relativistic single point energy calculations were conducted using PBE0 25% Hartree-Fock exchange) and BP86 functionals: the data are almost identical, and hence they are validated by the EPR calculations, but we report the BP86 data to enable like-for-like comparisons to previous work. The Quantum Theory of Atoms in Molecules analysis[17,18] was carried out within the ADF program. We quote Nalewajski-Mrozek bond orders since they reproduce expected bond multiplicities reliably in polar heavy atom structures whereas Mayer bond orders for polar bonds often do not always conform with chemical intuition.[19] The ADF-GUI (ADFview) was used to prepare the three-dimensional plots of the electron density.

FIGURES

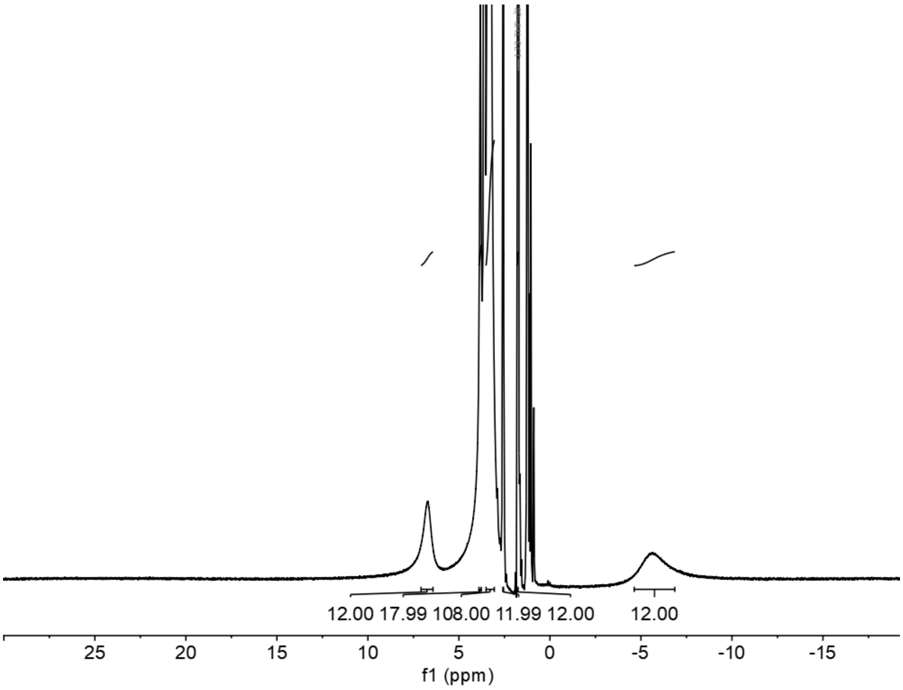

Figure S1. ^{1}H NMR (D_8-THF, 298 K) of **4**.

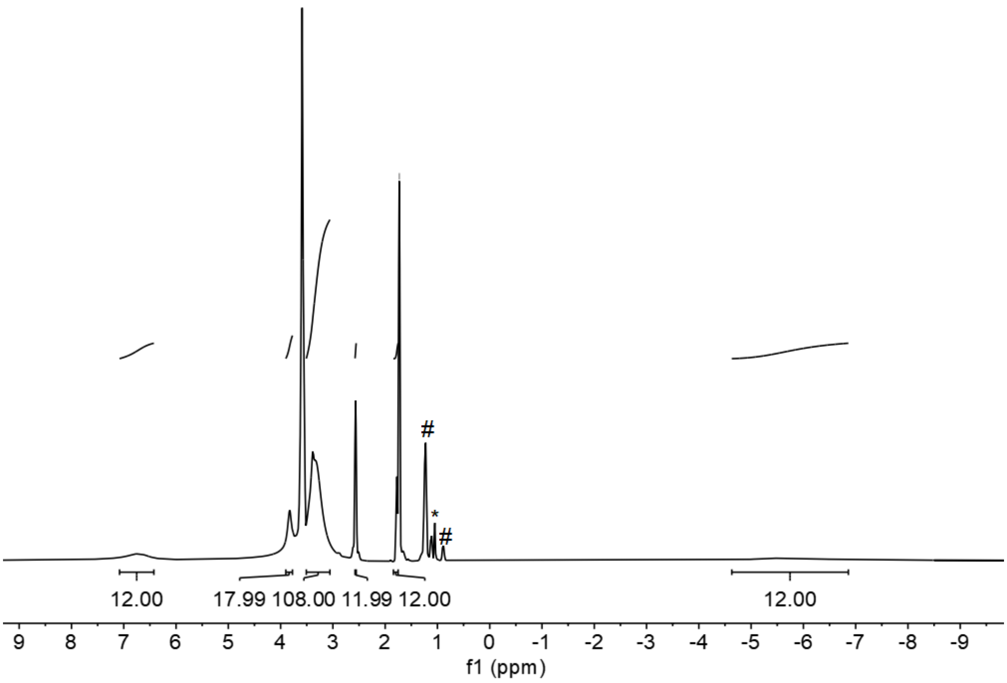

Figure S2. Zoom-in of the ^{1}H NMR (D_8-THF, 298 K) of **4**. # = trace pentane; * = trace diethyl ether. The resonance at 3.55 ppm assigned as the 12H $NCH_2C\mathit{H}_2OCH_2$ environment of the [K(2.2.2-cryptand)]$^+$ cation component is not integrated due to overlapping with the THF solvent resonance at ~3.58 ppm.

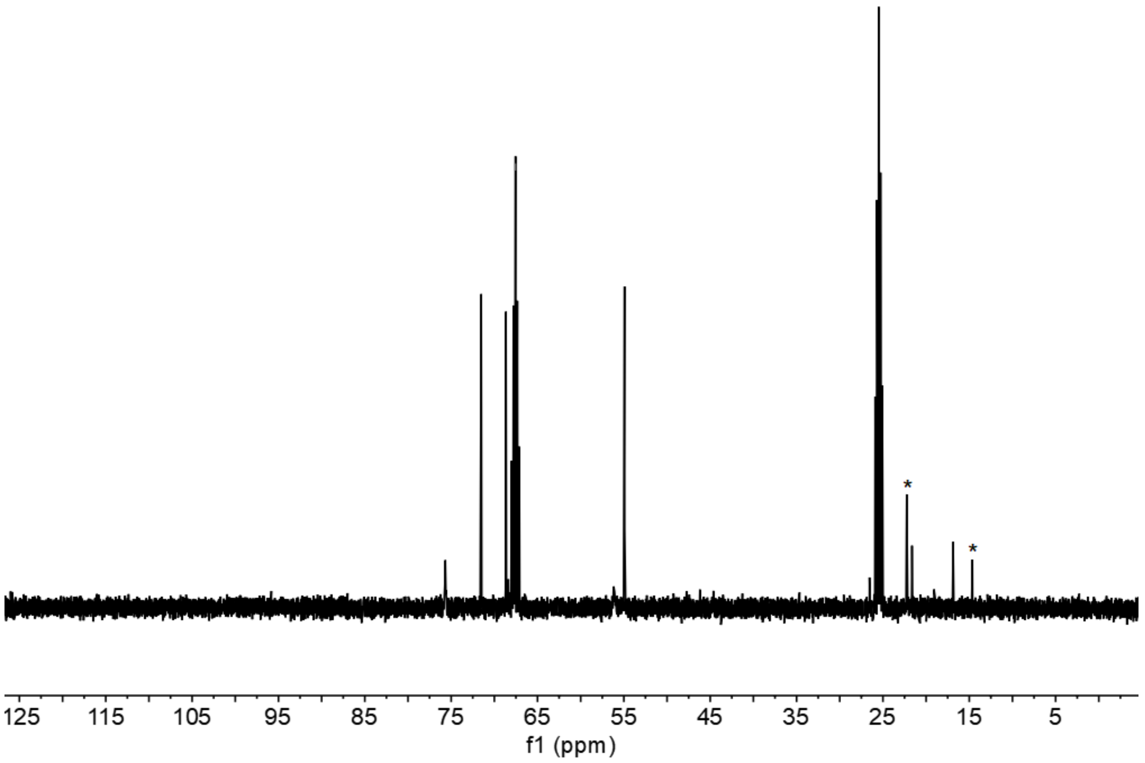

Figure S3. $^{13}C\{^{1}H\}$ NMR (D_8-THF, 298 K) of **4**. * = trace pentane.

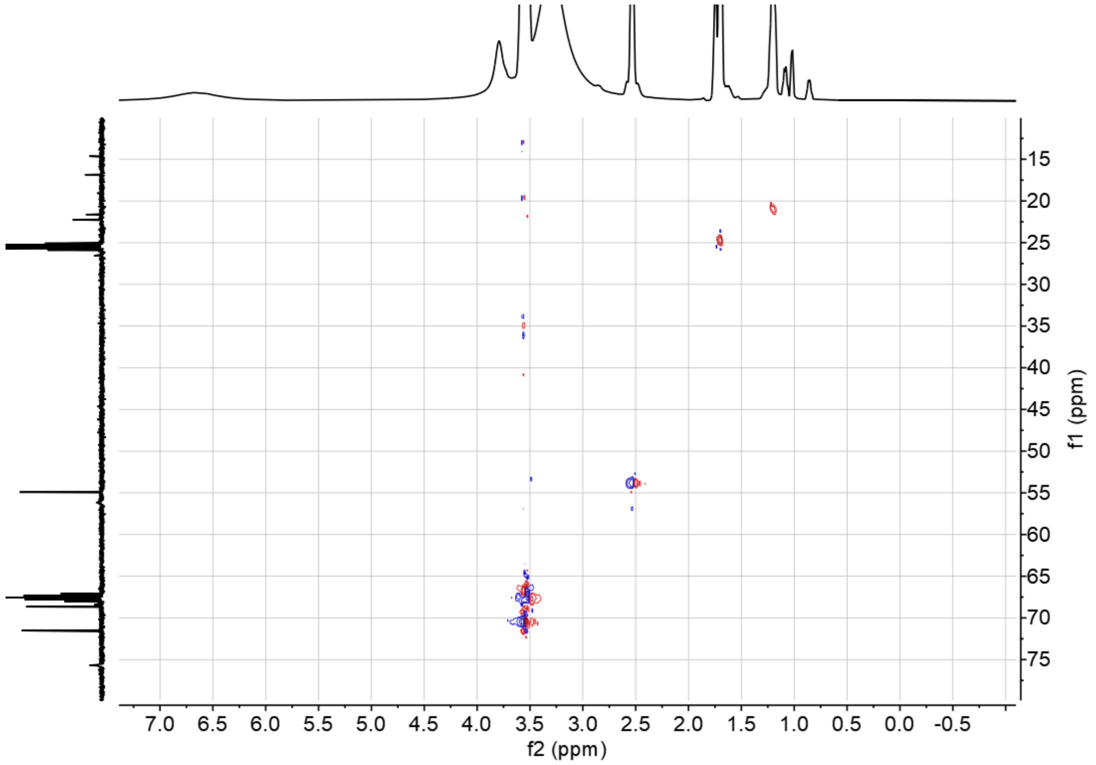

Figure S4. Selected region of ^{1}H-^{13}C HSQC NMR spectrum of **4** (D_8-THF, 298 K) showing correlations to the $[K(2.2.2\text{-cryptand})]^+$ cation component. No correlations were observed for the $[\{Th(Tren^{TIPS})\}_2(\mu\text{-}\eta^2{:}\eta^2\text{-}Sb_2)]^-$ anion presumably due to its paramagnetic S = ½ nature.

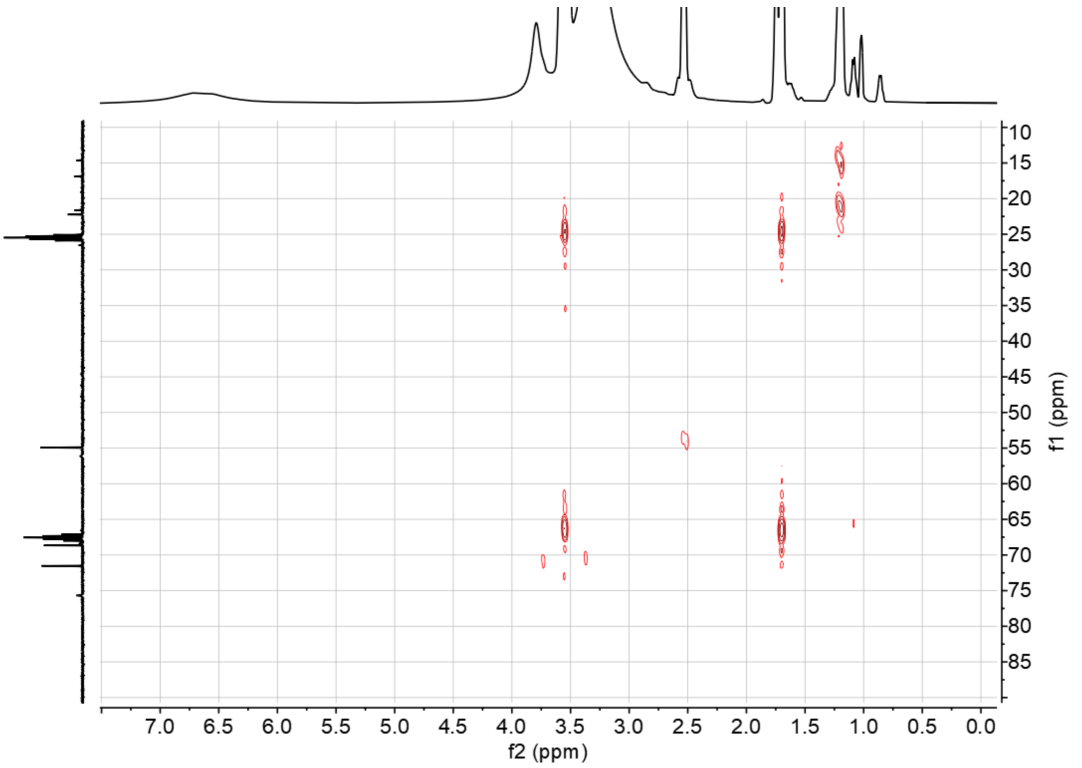

Figure S5. Selected region of ^{1}H-^{13}C HMBC NMR spectrum of **4** (D_8-THF, 298 K) showing correlations to the $[K(2.2.2\text{-cryptand})]^{+}$ cation component. No correlations were observed for the $[\{Th(Tren^{TIPS})\}_2(\mu\text{-}\eta^2\text{:}\eta^2\text{-}Sb_2)]^{-}$ anion presumably due to the paramagnetic (S = ½) nature of this component.

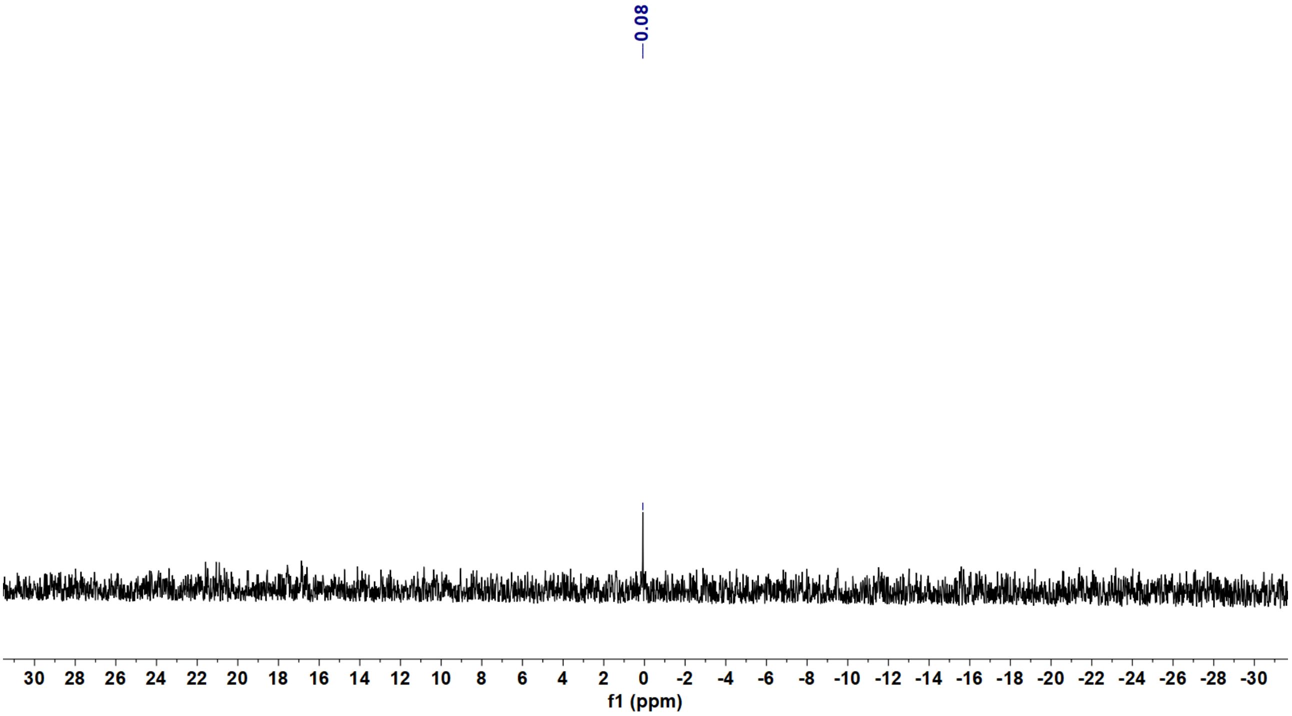

Figure S6. $^{29}Si\{^{1}H\}$ NMR (D_8-THF, 298 K) of **4**.

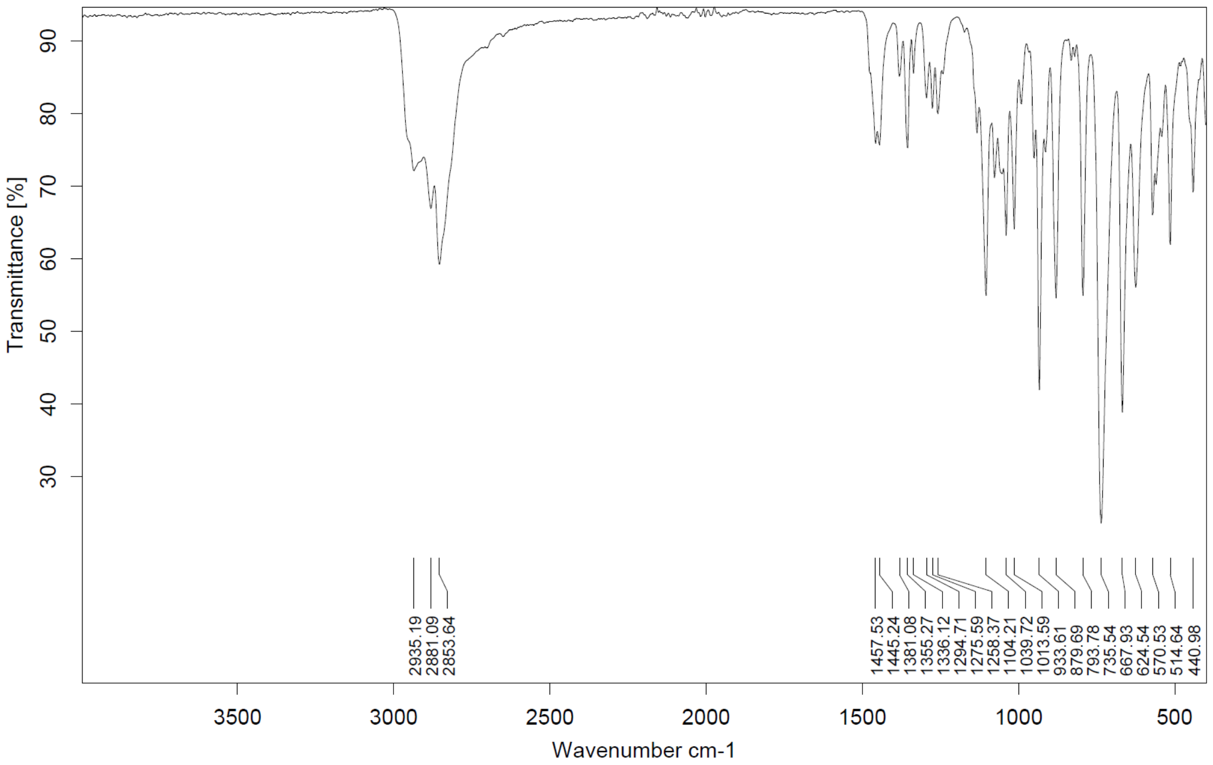

Figure S7. ATR-IR spectrum of **4**.

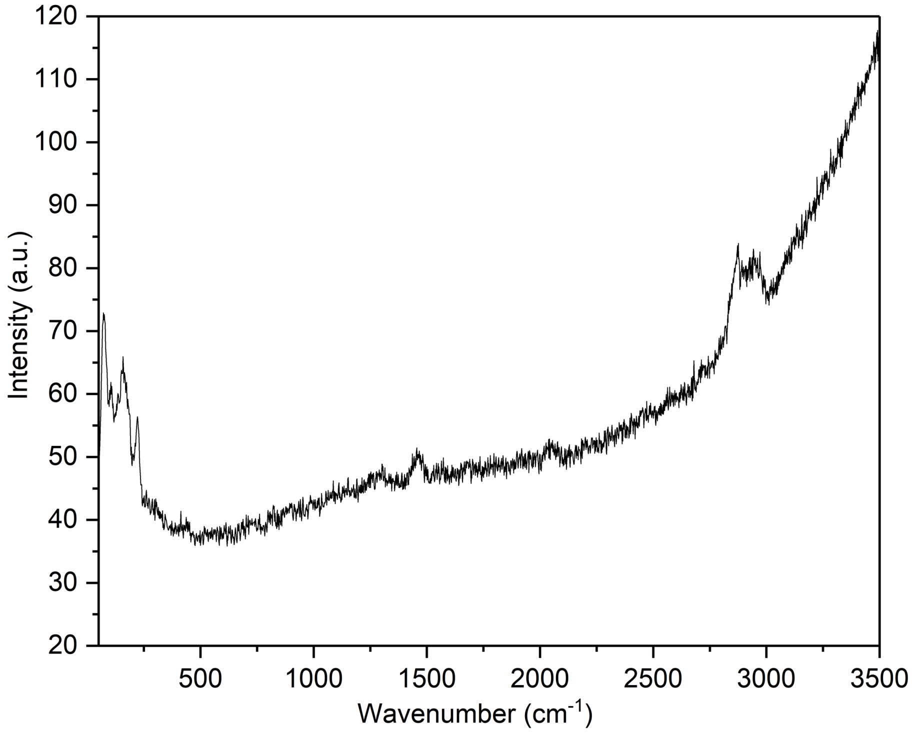

Figure S8. Raman spectrum of crystalline **4** over the range of 50 to 3500 cm^{-1}.

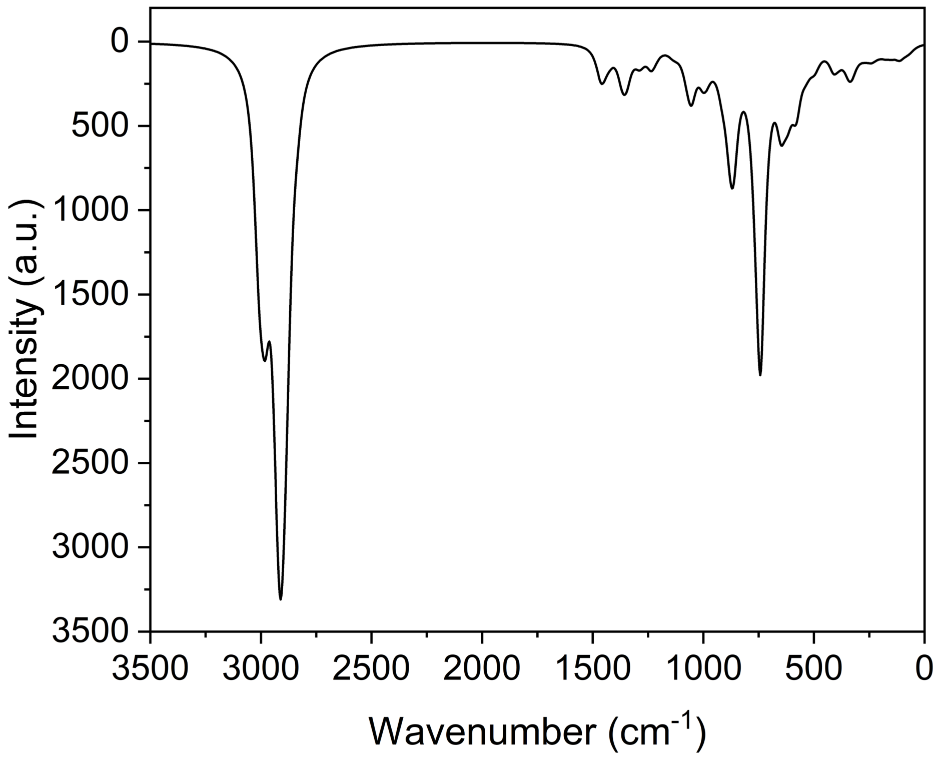

Figure S9. Computed vibrations of **4'** from an analytical frequencies DFT calculation, over the range 0-3600 cm^{-1}.

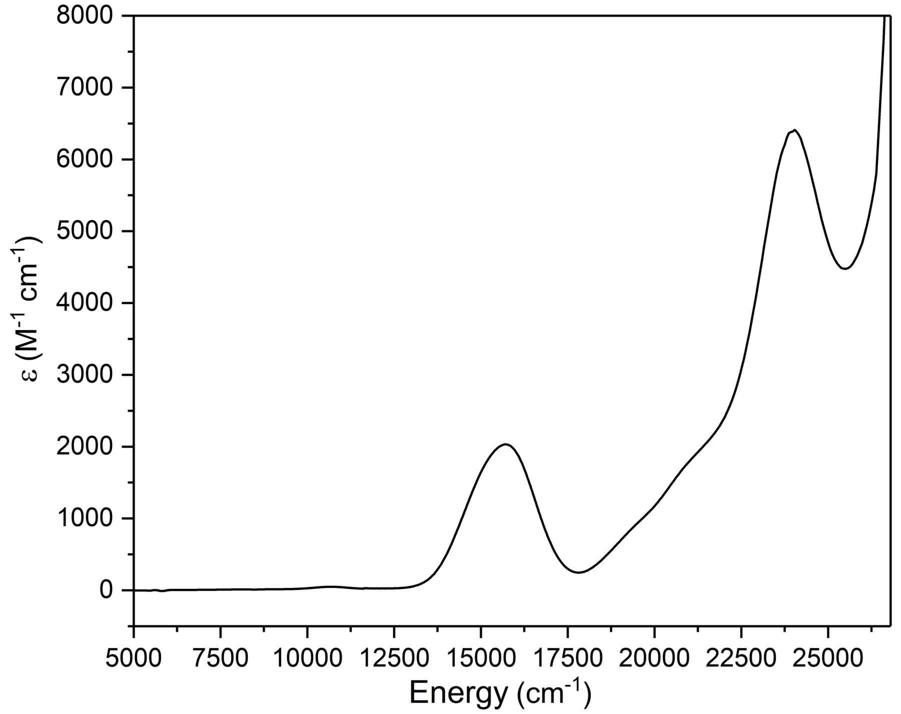

Figure S10. UV/vis/NIR spectra of **4** recorded in a saturated solution in THF (10 mM) over the range 5000-28000 cm^{-1}.

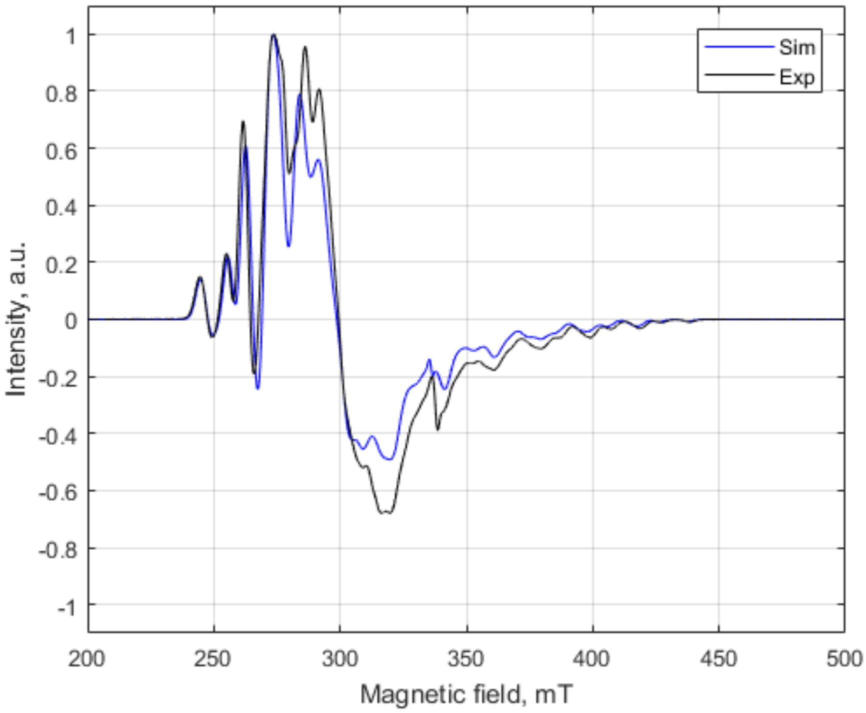

Figure S11. X-band EPR spectrum of powdered **4** at 5 K. The black line is the experimental data, the blue line is the simulated data. The data were simulated with a pseudo-axial spectrum with $g_x = 2.29$, $g_y = 2.26$, $g_z = 1.97$ ($g_{iso} = 2.18$) and $A_\perp$ and $A_{||}$ ($^{121/123}$Sb) values of 116, 162, and 538 MHz.

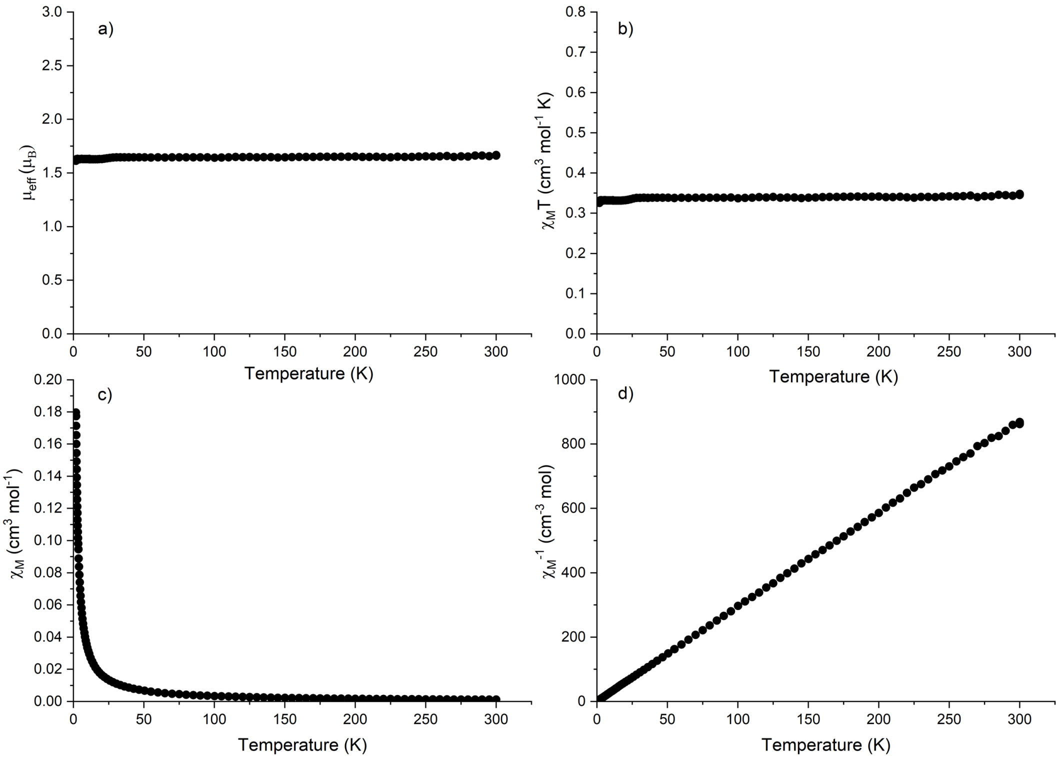

Figure S12. Variable-temperature SQUID magnetometry of **4** over the temperature range 1.8-300 K in an external 0.1 T field: a) μeff vs T; b) χ_MT vs T; c) χ_M vs T; d) χ_M^{-1} vs T.

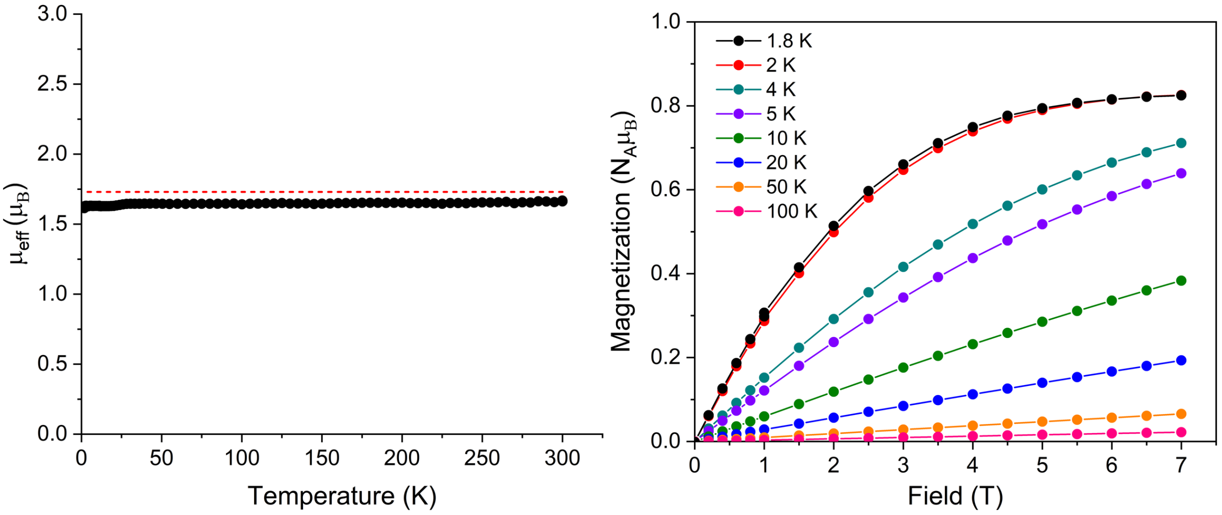

Figure S13. Temperature-dependent SQUID magnetometry data for **4**. Left: μ_{eff} (μ_B) vs Temperature (K) (black dots, experimental data; red dashes, a d^1 (S = ½) expected value for a $g = 2$ system; 300 K, 1.67 μ_B; 1.8 K, 1.61 μ_B). Right: Magnetization ($N_A\mu_B$) vs Field (Tesla) at 1.8, 2, 4, 5, 10, 20, 50, and 100 K (0.82 $N_A\mu_B$ at 7 T and 1.8 K).

TABLES

Table S1. XRD data for 4 and 5

	**4**	**5**
Formula	$C_{102}H_{222}K_2N_{12}O_{12}Sb_2Si_6Th_2$	$C_{120}H_{258}K_4N_{14}O_{18}Sb_6Si_6Th_2$
Fw, g mol^{-1}	2763.33	3704.91
Cryst size, mm	0.22 × 0.13 × 0.09	0.229 × 0.113 × 0.085
Crystal system	trigonal	orthorhombic
Space group	*R*-3m	*P*bcm
Collection Temperature (K)	110(2)	100(2)
a, (Å)	16.79860(10)	14.25300(10)
b, (Å)	16.79860(10)	25.0832(3)
c, (Å)	37.6236(3)	44.4819(4)
α, (°)	90	90
β, (°)	90	90
γ, (°)	120	90
V, (Å^3)	9194.69(13)	15902.8(3)
Z	2.99988	4
ρ_{calc} g cm^{-3}	1.497	1.547
μ, mm^{-1}	12.812	15.727
Reflections measured, unique, R_{int}	48586, 2476, 0.0677	143913, 17392, 0.1158
No. of reflections with $F^2 > 2\sigma(F^2)$	2452	15102
Transmission coefficient range	0.341-1.000	0.615-1.000
R, $R_w{}^a$ ($F^2 > 2\sigma(F^2)$)	0.0561, 0.1509	0.0861, 0.2338
R, $R_w{}^a$ (all data)	0.0564, 0.1511	0.0936, 0.2404
S^a, Parameters, Restraints	1.057, 221, 369	1.037, 1063, 1550
Max., min. difference map, e Å^{-3}	2.659, –3.432	2.935, –3.859

[a] Conventional $R = \Sigma||F_o| - |F_c||/\Sigma|F_o|$; $R_w = [\Sigma w(F_o^2 - F_c^2)^2/\Sigma w(F_o^2)^2]^{1/2}$; $S = [\Sigma w(F_o^2 - F_c^2)^2/\text{no. data} - \text{no. params})]^{1/2}$ for all data.

Table S2. Final coordinates and single point energy of geometry optimized 4'

1.C	-4.707209	-1.490677	-6.372688
2.C	-3.798102	1.878404	-5.045676
3.C	-2.789503	0.734647	-4.817259
4.C	-1.674369	-2.621520	-4.858391
5.C	-5.001986	-1.392211	-4.860750
6.C	1.772047	2.935868	-4.786105
7.C	4.826282	4.726497	-4.539405
8.C	5.594345	2.320923	-4.547191
9.C	-1.494887	1.286241	-4.213266
10.C	3.885795	-3.478721	-4.164530
11.C	-5.619466	-2.714904	-4.361910
12.C	2.447604	0.662924	-3.915780
13.C	5.051125	3.470513	-3.670758
14.C	-2.164643	-2.042357	-3.516745
15.C	2.155419	2.122542	-3.533837
16.C	1.563365	-3.186532	-3.244645
17.C	2.899953	-3.912895	-3.061185
18.C	-2.559331	-3.180508	-2.557928
19.C	-5.512561	0.363506	-2.399805
20.C	4.915754	-6.293155	-1.956837
21.C	2.926600	4.670189	-1.853505
22.C	-6.135068	0.882623	-1.113891
23.C	5.173237	-4.931730	-1.274550
24.C	-2.677563	3.994164	-1.456913
25.C	5.526656	2.243442	-0.904169
26.C	5.548706	-1.916049	-1.494000
27.C	6.141282	-0.530921	-1.323972
28.C	1.564820	4.659280	-1.146115
29.C	3.967901	5.376685	-0.961360
30.C	6.132325	1.399483	0.201049
31.C	-5.793712	5.158457	-0.116792
32.C	1.903016	-5.769559	-0.169949
33.C	5.793712	-5.158457	0.116792
34.C	2.323947	-4.293749	-0.008591
35.C	-1.903016	5.769559	0.169949
36.C	-2.323947	4.293749	0.008591
37.C	-6.132325	-1.399483	-0.201049
38.C	-3.967901	-5.376685	0.961360
39.C	-6.141282	0.530921	1.323972
40.C	-5.173237	4.931730	1.274550
41.C	-5.548706	1.916049	1.494000
42.C	-1.564820	-4.659280	1.146115
43.C	-5.526656	-2.243442	0.904169
44.C	6.135068	-0.882623	1.113891
45.C	2.677563	-3.994164	1.456913
46.C	-4.915754	6.293155	1.956837
47.C	-2.926600	-4.670189	1.853505
48.C	5.512561	-0.363506	2.399805
49.C	2.559331	3.180508	2.557928

50.C	-2.899953	3.912895	3.061185
51.C	-5.051125	-3.470513	3.670758
52.C	-1.563365	3.186532	3.244645
53.C	-2.155419	-2.122542	3.533837
54.C	2.164643	2.042357	3.516745
55.C	5.619466	2.714904	4.361910
56.C	-2.447604	-0.662924	3.915780
57.C	-5.594345	-2.320923	4.547191
58.C	-4.826282	-4.726497	4.539405
59.C	-3.885795	3.478721	4.164530
60.C	5.001986	1.392211	4.860750
61.C	1.494887	-1.286241	4.213266
62.C	-1.772047	-2.935868	4.786105
63.C	1.674369	2.621520	4.858391
64.C	2.789503	-0.734647	4.817259
65.C	3.798102	-1.878404	5.045676
66.C	4.707209	1.490677	6.372688
67.H	-5.611744	-1.817465	-6.918195
68.H	-4.399055	-0.526026	-6.800189
69.H	-3.913643	-2.219306	-6.599832
70.H	-3.374382	2.649790	-5.712566
71.H	-2.544863	0.303741	-5.810165
72.H	-4.741795	1.535136	-5.495154
73.H	-1.342762	-1.840013	-5.559516
74.H	2.592326	2.962095	-5.520212
75.H	-2.466118	-3.202041	-5.357382
76.H	0.902811	2.484559	-5.290616
77.H	3.466253	-3.677997	-5.168032
78.H	3.999773	4.603901	-5.256327
79.H	5.735479	4.950732	-5.130124
80.H	-1.137557	2.163370	-4.781684
81.H	6.537472	2.612442	-5.041918
82.H	4.881979	2.054850	-5.343366
83.H	-6.556559	-2.942687	-4.900821
84.H	-5.785060	-0.620840	-4.766311
85.H	-0.826153	-3.303695	-4.700549
86.H	1.507217	3.976341	-4.544664
87.H	3.321496	0.571172	-4.577720
88.H	4.853812	-3.996905	-4.102859
89.H	-4.038961	2.377660	-4.094041
90.H	-0.687368	0.541570	-4.208145
91.H	1.588336	0.213808	-4.439135
92.H	-4.936754	-3.562314	-4.531939
93.H	4.076902	-2.396632	-4.107958
94.H	1.205957	-3.288967	-4.283935
95.H	4.606759	5.615998	-3.933587
96.H	5.790612	1.406086	-3.969411
97.H	-1.632878	1.611899	-3.170220
98.H	-5.850210	-2.697587	-3.286756
99.H	2.711310	-4.997817	-3.190197
100.H	-5.605971	1.155892	-3.152385

101.H	1.654930	-2.109969	-3.035156
102.H	4.606205	-6.181878	-3.004703
103.H	-1.312508	-1.514312	-3.044185
104.H	5.858948	3.743230	-2.972236
105.H	2.649352	0.023683	-3.040224
106.H	1.276628	2.104765	-2.859201
107.H	-3.383012	-3.790160	-2.960319
108.H	2.811050	5.281050	-2.770582
109.H	0.779834	-3.570638	-2.577697
110.H	-6.151123	-0.466530	-2.760764
111.H	-1.703118	-3.852222	-2.385761
112.H	5.836461	-6.904970	-1.948854
113.H	6.174249	2.132272	-1.794796
114.H	5.656834	-2.167896	-2.572231
115.H	5.942130	-4.435733	-1.892903
116.H	5.723771	0.148535	-2.070212
117.H	-1.825250	4.221517	-2.117895
118.H	-7.236773	0.957791	-1.220909
119.H	4.136181	-6.877642	-1.443623
120.H	-3.535263	4.589545	-1.806723
121.H	0.766321	4.251274	-1.784427
122.H	4.950657	5.476523	-1.449223
123.H	-5.736052	1.881729	-0.895269
124.H	-2.872713	-2.812922	-1.570184
125.H	-2.926699	2.934169	-1.614905
126.H	5.635419	3.296975	-0.597949
127.H	7.234562	1.524536	0.223843
128.H	1.628710	-6.017764	-1.206518
129.H	-5.120552	5.745713	-0.762212
130.H	7.242267	-0.560427	-1.438246
131.H	1.270883	5.683974	-0.854919
132.H	3.629618	6.392875	-0.690406
133.H	-6.007808	4.218005	-0.647645
134.H	-1.026784	5.987186	-0.461703
135.H	-5.724040	-1.711793	-1.169999
136.H	-6.742169	5.721811	-0.043641
137.H	6.210025	-2.626382	-0.971669
138.H	1.586219	4.046983	-0.234509
139.H	-2.703762	6.457859	-0.138464
140.H	4.112258	4.825345	-0.020880
141.H	6.742169	-5.721811	0.043641
142.H	1.437535	-3.675097	-0.246109
143.H	2.703762	-6.457859	0.138464
144.H	-4.112258	-4.825345	0.020880
145.H	6.007808	-4.218005	0.647645
146.H	5.724040	1.711793	1.169999
147.H	-6.210025	2.626382	0.971669
148.H	-1.437535	3.675097	0.246109
149.H	-1.586219	-4.046983	0.234509
150.H	-3.629618	-6.392875	0.690406
151.H	1.026784	-5.987186	0.461703

152.H	-7.242267	0.560427	1.438246
153.H	5.120552	-5.745713	0.762212
154.H	-7.234562	-1.524536	-0.223843
155.H	-1.270883	-5.683974	0.854919
156.H	-1.628710	6.017764	1.206518
157.H	-4.136181	6.877642	1.443623
158.H	-5.635419	-3.296975	0.597949
159.H	-4.950657	-5.476523	1.449223
160.H	5.736052	-1.881729	0.895269
161.H	-5.836461	6.904970	1.948854
162.H	7.236773	-0.957791	1.220909
163.H	-5.942130	4.435733	1.892903
164.H	2.926699	-2.934169	1.614905
165.H	3.535263	-4.589545	1.806723
166.H	2.872713	2.812922	1.570184
167.H	-5.723771	-0.148535	2.070212
168.H	-0.766321	-4.251274	1.784427
169.H	-5.656834	2.167896	2.572231
170.H	1.825250	-4.221517	2.117895
171.H	-6.174249	-2.132272	1.794796
172.H	-4.606205	6.181878	3.004703
173.H	1.703118	3.852222	2.385761
174.H	6.151123	0.466530	2.760764
175.H	-2.811050	-5.281050	2.770582
176.H	-5.858948	-3.743230	2.972236
177.H	3.383012	3.790160	2.960319
178.H	-0.779834	3.570638	2.577697
179.H	5.850210	2.697587	3.286756
180.H	-2.711310	4.997817	3.190197
181.H	-1.276628	-2.104765	2.859201
182.H	5.605971	-1.155892	3.152385
183.H	-2.649352	-0.023683	3.040224
184.H	1.312508	1.514312	3.044185
185.H	-1.654930	2.109969	3.035156
186.H	-5.790612	-1.406086	3.969411
187.H	-4.606759	-5.615998	3.933587
188.H	1.632878	-1.611899	3.170220
189.H	4.936754	3.562314	4.531939
190.H	-4.853812	3.996905	4.102859
191.H	-4.076902	2.396632	4.107958
192.H	6.556559	2.942687	4.900821
193.H	4.038961	-2.377660	4.094041
194.H	-1.205957	3.288967	4.283935
195.H	5.785060	0.620840	4.766311
196.H	-5.735479	-4.950732	5.130124
197.H	-6.537472	-2.612442	5.041918
198.H	-3.321496	-0.571172	4.577720
199.H	-1.507217	-3.976341	4.544664
200.H	-1.588336	-0.213808	4.439135
201.H	0.687368	-0.541570	4.208145
202.H	0.826153	3.303695	4.700549

203.H	-4.881979	-2.054850	5.343366
204.H	-3.999773	-4.603901	5.256327
205.H	-3.466253	3.677997	5.168032
206.H	2.466118	3.202041	5.357382
207.H	1.137557	-2.163370	4.781684
208.H	-2.592326	-2.962095	5.520212
209.H	-0.902811	-2.484559	5.290616
210.H	4.741795	-1.535136	5.495154
211.H	1.342762	1.840013	5.559516
212.H	3.374382	-2.649790	5.712566
213.H	2.544863	-0.303741	5.810165
214.H	3.913643	2.219306	6.599832
215.H	5.611744	1.817465	6.918195
216.H	4.399055	0.526026	6.800189
217.N	-4.107621	-0.097219	-2.206621
218.N	4.145895	-2.020307	-1.017719
219.N	4.119103	1.872933	-1.213316
220.N	5.752692	-0.007485	0.000748
221.N	-5.752692	0.007485	-0.000748
222.N	-4.119103	-1.872933	1.213316
223.N	-4.145895	2.020307	1.017719
224.N	4.107621	0.097219	2.206621
225.Sb	0.021137	1.362736	-0.152880
226.Sb	-0.021137	-1.362736	0.152880
227.Si	-3.516457	-0.711224	-3.774046
228.Si	3.545318	2.959872	-2.511037
229.Si	3.630101	-3.715287	-1.293044
230.Si	-3.630101	3.715287	1.293044
231.Si	-3.545318	-2.959872	2.511037
232.Si	3.516457	0.711224	3.774046
233.Th	-3.150115	0.018777	0.009655
234.Th	3.150115	-0.018777	-0.009655

Energy: −1257.05348601 eV

Table S3. TD-DFT Absorptions for 4′ with hydrogen atoms omitted for clarity and only the principal (>40%) virtual orbital contribution depicted.

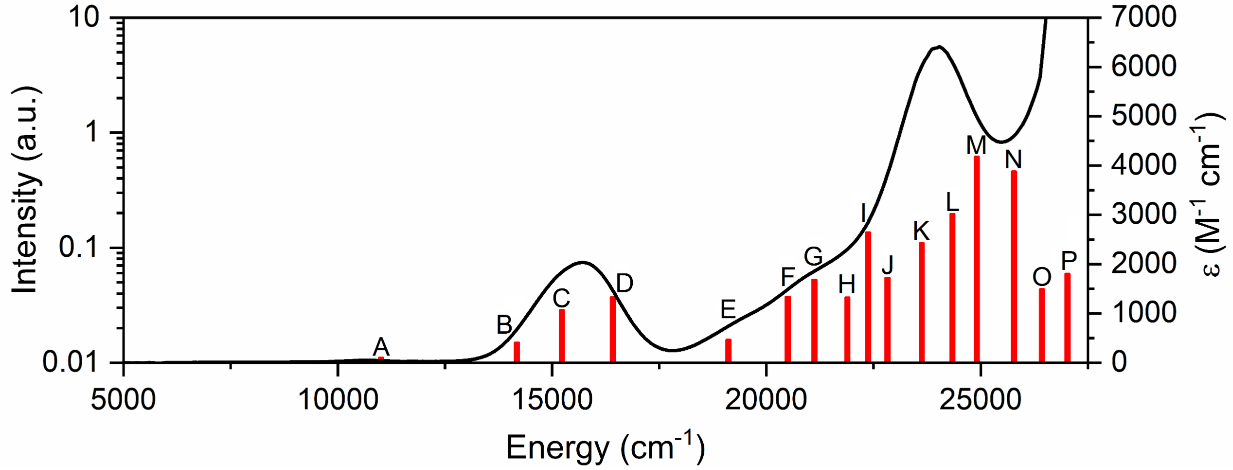

Peak	Energy (cm^{-1})	Donor orbital (occupied)	Acceptor orbital (unoccupied)	Assignment
**A**	11210	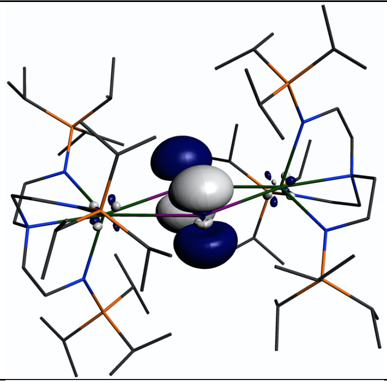	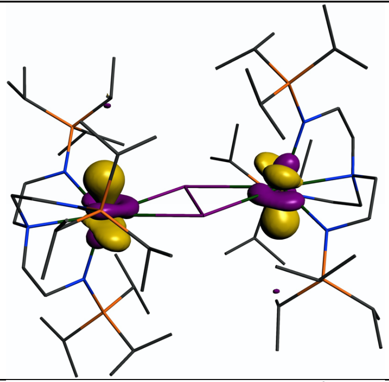	$B(\pi_{g\perp})$ π^*-f
**B**	14178	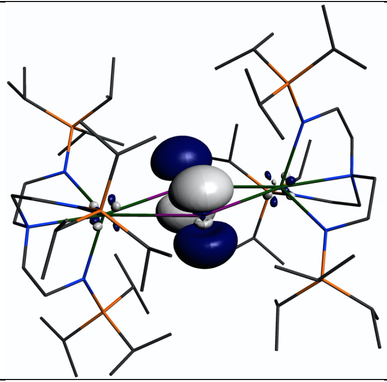	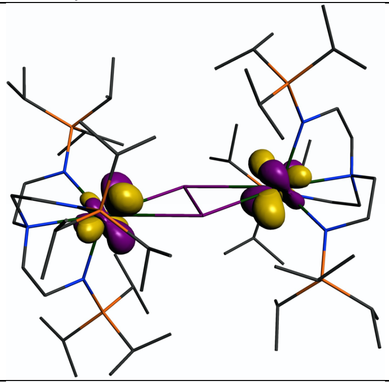	$B(\pi_{g\perp})$ π^*-f
**C**	15228	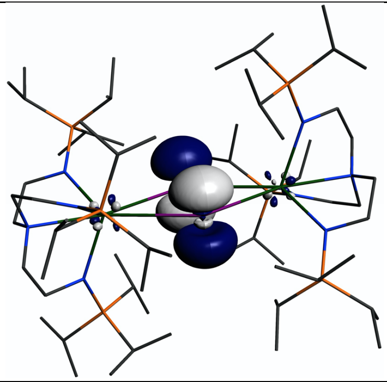	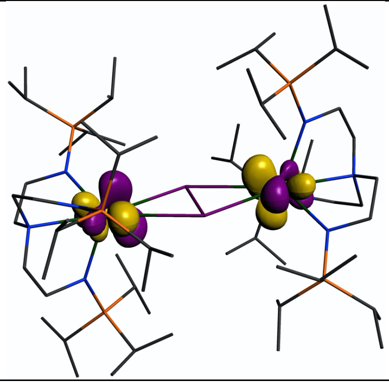	$B(\pi_{g\perp})$ π^*-f

**D**	16414	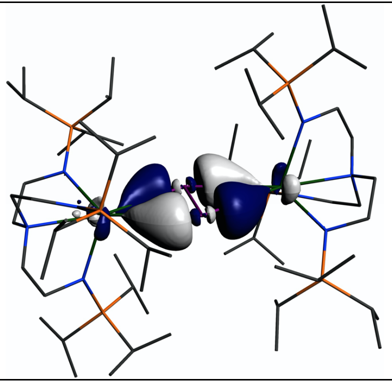	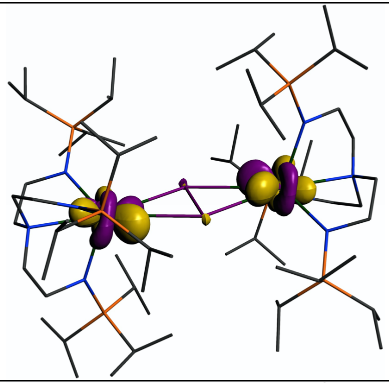	A($\pi_{g=}$) π^*-f
**E**	19112	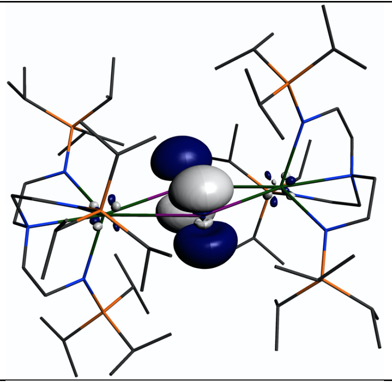	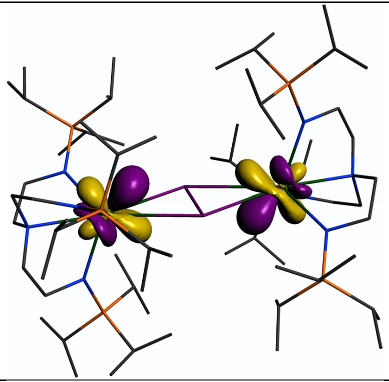	B($\pi_{g\perp}$) π^*-f/d
**F**	20500	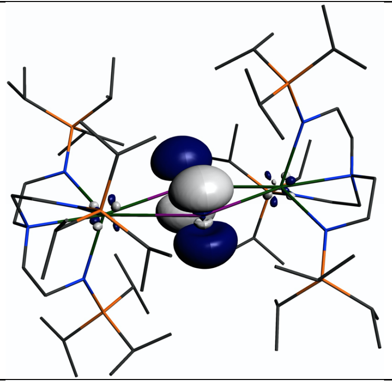	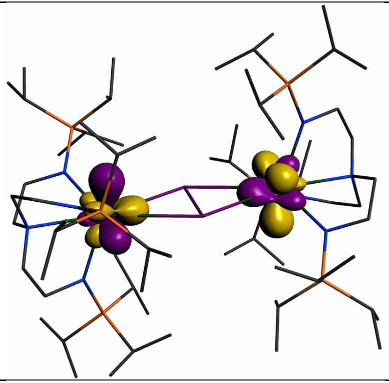	B($\pi_{g\perp}$) π^*-f/d
**G**	21120	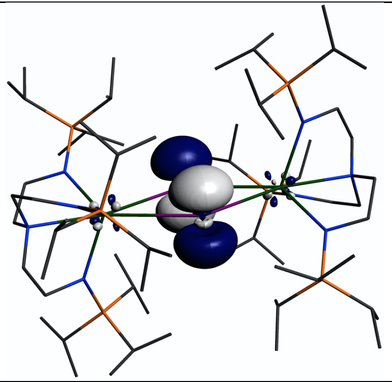	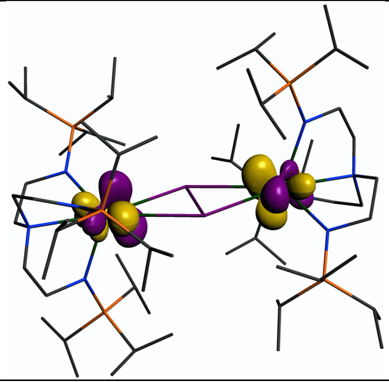	B($\pi_{g\perp}$) π^*-f
**H**	21886	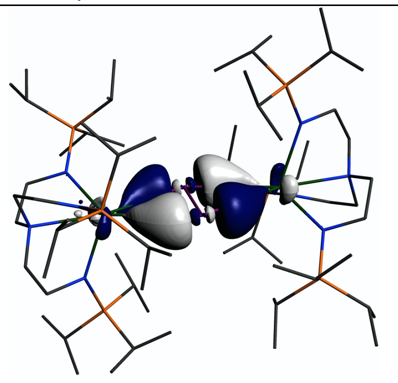	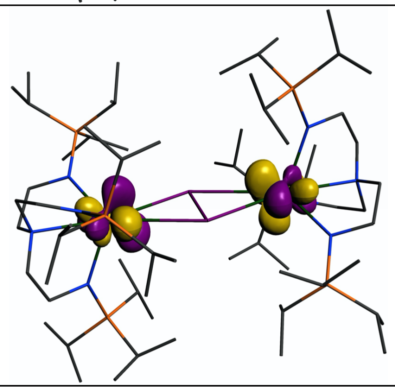	A($\pi_{g=}$) π^*-f

**I**	22374	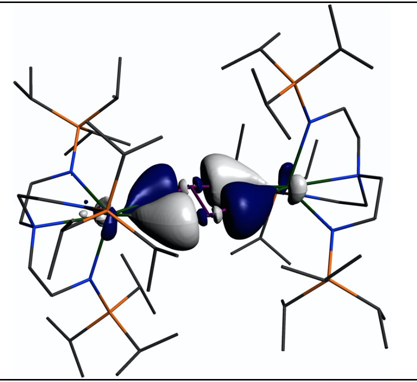	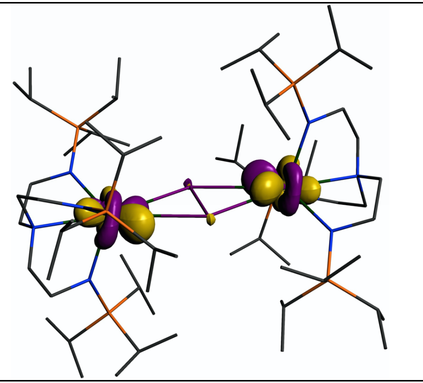	A($\pi_{g=}$) π^*-f
**J**	22826	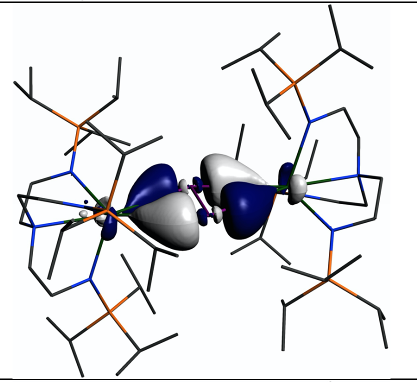	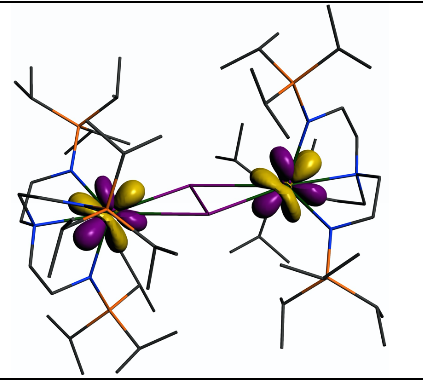	A($\pi_{g=}$) π^*-f
**K**	23624	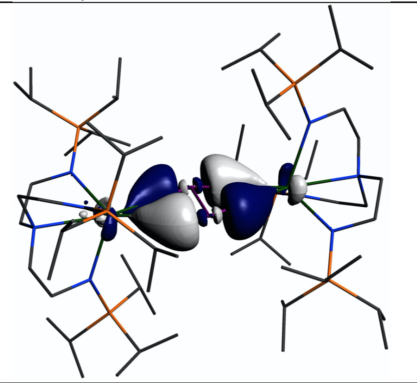	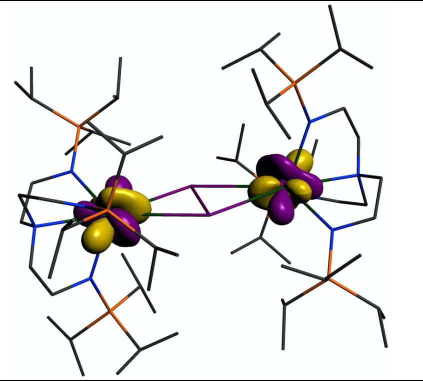	A($\pi_{g=}$) π^*-f
**L**	24341	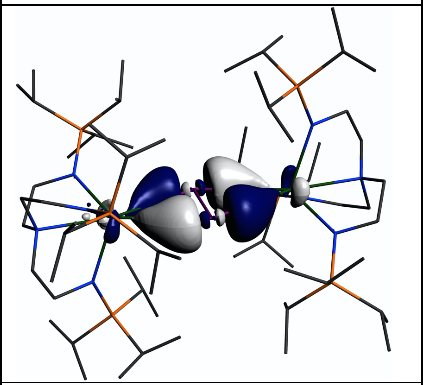	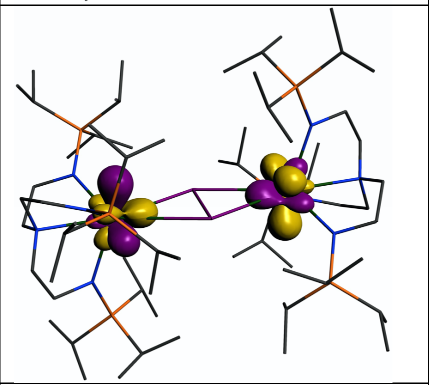	A($\pi_{g=}$) π^*-f/d
**M**	24910	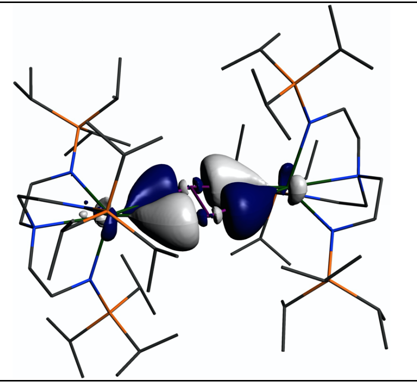	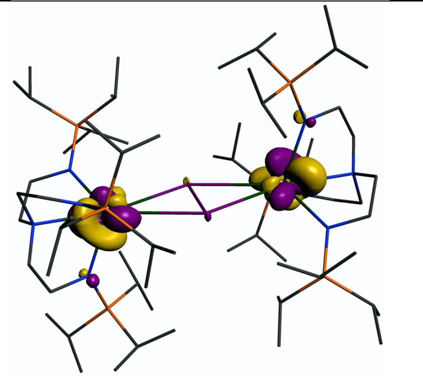	A($\pi_{g=}$) π^*-d/f

**N**	25775	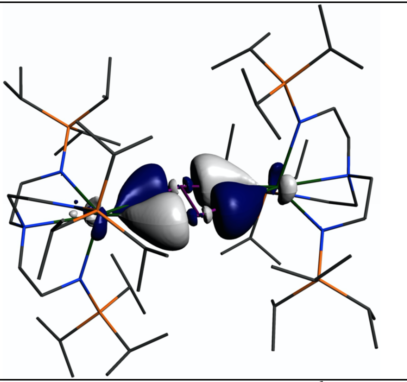	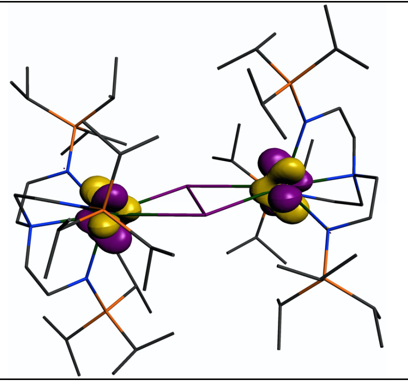	$A(\pi_{g=})$ π*-f
**O**	26429	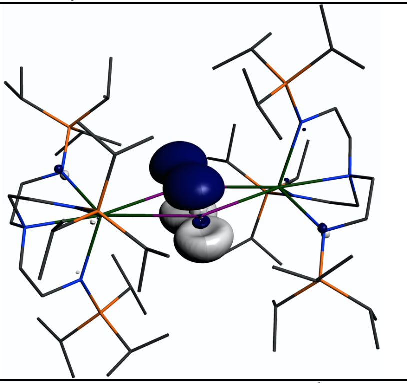	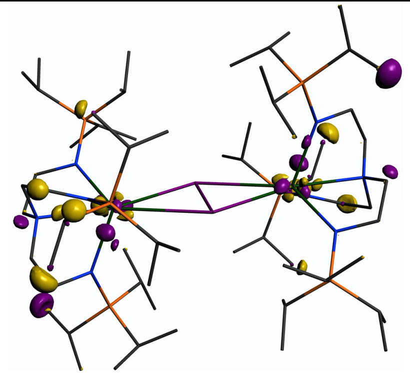	$A(\pi_{u\perp})$ π-d
**P**	27023	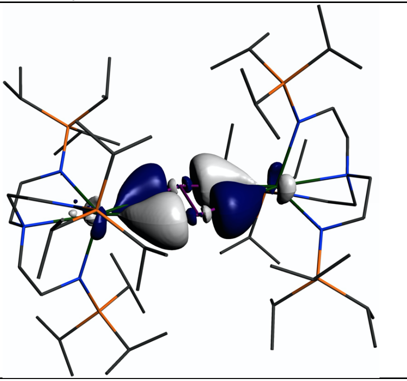	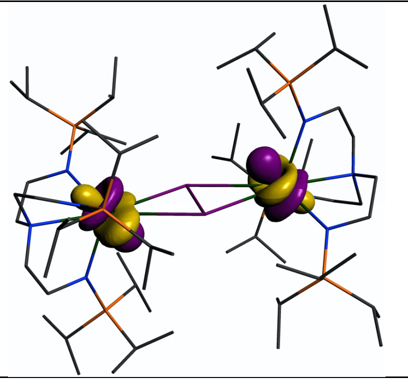	$A(\pi_{g=})$ π*-f/d

REFERENCES

1. Bergbreiter, D. E.; Killough, J. M. Reactions of potassium-graphite *J. Am. Chem. Soc.* **1978**, *100*, 2126-2134.
2. Du, J.; Dollberg, K.; Seed, J. A.; Wooles, A. J.; Von Hänisch, C.; Liddle, S. T. Thorium(IV)-antimony complexes exhibiting single, double, and triple polar covalent metal-metal bonds. *Nat. Chem.* **2024**, *16*, 780-790.
3. Sheldrick, G. M. SHELXT - Integrated space-group and crystal-structure determination. *Acta Cryst. Sect. A* **2015**, *71*, 3-8.
4. CrysAlisPRO version 40.69, Oxford Diffraction /Agilent Technologies UK Ltd, Yarnton, England.
5. Sheldrick, G. M. Crystal structure refinement with SHELXL. *Acta Cryst. Sect. C* **2015**, *71*, 3-8.
6. Dolomanov, O. V.; Bourhis, L. J.; Gildea, R. J.; Howard, J. A. K.; Puschmann, H. OLEX2: a complete structure solution, refinement and analysis program. *J. Appl. Cryst.* **2009**, *42*, 339-341.
7. Farugia, L. J. WinGX and ORTEP for Windows: an update. *J. Appl. Cryst.* **2012**, *45*, 849-854.
8. Persistence of Vision (TM) Raytracer, Persistence of Vision Pty. Ltd., Williamstown, Victoria, Australia.
9. Fonseca Guerra, C.; Snijders, J. G.; Te Velde, G.; Baerends, E. J. Towards an order-N DFT Method. *Theor. Chem. Acc.* **1998**, *99*, 391-403.
10. Te Velde, G.; Bickelhaupt, F. M.; Baerends, E. J.; Fonseca Guerra, C.; Van Gisbergen, S. J. A.; Snijders, J. G.; Ziegler, T. Chemistry with ADF. *J. Comput. Chem.* **2001**, *22*, 931-967.
11. Van Lenthe, E.; Baerends, E. J.; Snijders, J. G. Relativistic regular two-component Hamiltonians. *J. Chem. Phys.* **1993**, *99*, 4597-4610.
12. Van Lenthe, E.; Baerends, E. J.; Snijders, J. G. Relativistic total energy using regular approximations. *J. Chem. Phys.* **1994**, *101*, 9783-9792.

13. Van Lenthe, E.; Ehlers, A. E.; Baerends, E. J. Geometry optimization in the Zero Order Regular Approximation for relativistic effects. *J. Chem. Phys.* **1999**, *110*, 8943-8953.

14. Vosko, S. H.; Wilk, L.; Nusair, M. Accurate spin-dependent electron liquid correlation energies for local spin density calculations: a critical analysis. *Can. J. Phys.* **1980**, *58*, 1200-1211.

15. Becke, A. D. Density-functional exchange-energy approximation with correct asymptotic behaviour. *Phys. Rev. A.* **1988**, *38*, 3098-3100.

16. Perdew, J. P. Density-functional approximation for the correlation energy of the inhomogeneous electron gas. *Phys. Rev. B.* **1986**, *33*, 8822-8824.

17. Bader, R. F. W. *Atoms in Molecules: A Quantum Theory*, Oxford University Press, New York, 1990.

18. Bader, R. F. W. A bond path: a universal indicator of bonded interactions. *J. Phys. Chem. A* **1998**, *102*, 7314-7323.

19. Motta, L. C.; Autschbach, J. Actinide inverse trans influence versus cooperative pushing from below and multi-center bonding. *Nat. Commun.* **2023**, *14*, 4307.
